# The implications of Industry 4.0 on supply chains amid the COVID-19 pandemic: a systematic review

**DOI:** 10.12688/f1000research.73138.1

**Published:** 2021-10-05

**Authors:** Mohammad Nurul Hassan Reza, Sreenivasan Jayashree, Chinnasamy Agamudai Nambi Malarvizhi, Md Abdur Rauf, Kalaivani Jayaraman, Syed Hussain Shareef

**Affiliations:** 1 Faculty of Management, Multimedia University, Cyberjaya, Selangor, 63100, Malaysia; 2 Faculty of Educational Study, University Putra Malaysia, Serdang, Malaysia, 43400, Malaysia; 3 Faculty of Accountancy and Business, Universiti Tunku Abdul Rahman, Bandar Sungai Long, Selangor, 43000, Malaysia; 4 NTT DATA Business Solutions, Cyberjaya, Selangor, 63000, Malaysia

**Keywords:** Industry 4.0, emerging technologies, supply chain, COVID 19, Systematic Literature Review

## Abstract

**Background**: COVID-19 has caused significant disruptions in supply chains. It has increased the demand for products and decreased the supply of raw materials. This has interrupted many production processes. The emerging technologies of Industry 4.0 have the potential to streamline supply chains by improving time-sensitive customized solutions during this emergency.

**Purpose**: This research examines the effects of the epidemic on supply chains and how these effects are reduced through Industry 4.0 technology.

**Design/methodology/approach:** An extensive literature review using the “Preferred Reporting Items for Systematic Review and Meta-Analysis” method was carried out on the impact of the COVID-19 pandemic on supply chains and Industry 4.0 technologies. The study was undertaken by selecting keywords validated by experts and a search was conducted in the Scopus, ProQuest, and Google Scholar databases. Publications from the leading journals on these topics were selected. The bibliographical search resulted in 1484 articles followed by multiple layers of filtering. Finally, the most pertinent articles were selected for reviewing, and a total of 53 articles were analysed.

**Findings:** This study discusses the impact of COVID-19 on the supply chain and how the emerging technologies of Industry 4.0 can help manufacturers to ease the impact. These technologies will enhance the production system through the automation and optimization of production flow convergence, enabling efficiencies and improvements among the suppliers, manufacturers, and consumers in the COVID-19 situation.

**Originality/value:** The study summarizes the impact of the COVID-19 on supply chains and shows the potential of Industry 4.0 technologies to lessen the impact on manufacturing supply chains. This is valuable information for policymakers and practitioners so that they can get insights and take necessary actions.

## Introduction

The COVID-19 pandemic has already had a crucial impact on human health as well as countries’ economies. Supply chains in various industries have been under tremendous pressure to avoid considerable disruptions in their operations. ^3^ COVID-19 has also affected every member of the supply chain process. ^56, 58^ The failure of many nations and businesses to deal with the COVID-19 pandemic is attributable to their supply chains and their inability to deliver products and services. ^63^ Supply chain issues, those associated with sourcing techniques, have created substantial disruptions in various supply chains. Lack of risk management, adoption of the single-sourcing strategy, and supplier delivery delays are examples. ^64^ These distractions have generated numerous lessons in understanding supply chain management, advising both researchers and practitioners to reconsider how supply chain strategies should address new disruptive threats. ^3^ In this regard, the role of Industry 4.0 technologies in supply chain management and their revival during COVID-19 is explored in this article.

Industry 4.0 fosters decentralized manufacturing systems enabled by technological innovations. ^65^ The concept offers a business atmosphere that integrates humans, machines, equipment, and operational processes through Cyber-Physical Systems and the Internet. ^66^ Industry 4.0 integrates its emerging technologies into the entire organizational setting, facilitates automated and dynamic production systems, ^67^ significantly improves the quality of products and services by digitizing the operational activities. ^68^ However, the COVID-19 pandemic emerged when the supply chains had been called upon to transform and adapt the dynamics of Industry 4.0. Incorporating Industry 4.0 technologies has become a strategic imperative for supply chains to improve their competitiveness in the market. ^1, 4^ These technologies play a significant role in optimizing the performance of supply chain operations for better results. ^6^ Many academics believe that Industry 4.0 technologies, such as the Internet of Things, ^5, 9, 37^ big data, ^14, 15, 21, 38^ cloud computing, ^22, 23^ additive manufacturing, ^48, 60^ and blockchain, ^17, 19, 20^ can encourage supply chains in times of crisis, and they call for more research in this area. ^43, 45, 69^ Previous studies on epidemics did not address the employment of emerging technologies in the recovery process ^61^ as well as the impact on commercial supply chains. ^70^ Consequently, it is unclear how a supply chain can utilise technologies to increase flexibility and response time. ^58^ This requires a holistic approach. ^71^ Furthermore, existing literature lacks a comprehensive review on the role of new technologies in enabling supply chains, especially in emergencies such as the COVID-19 pandemic. ^3, 58^ Therefore, Chowdhury, Paul, Kaisar and Moktadir ^58^ and Frederico ^3^ suggested looking into the role of emerging technologies of Industry 4.0 in regulating the effects of COVID-19. The current study has conducted a systematic literature review to close this gap. Also, the study assesses the overall role of Industry 4.0 technologies in developing a holistic supply chain framework and focuses on the potential applications of the emerging technologies to address pandemic-related supply chain problems.

Hence a comprehensive literature review was conducted on Industry 4.0 technologies, supply chain, and COVID-19 for exploratory analysis and a deeper understanding to answer the following research queries:

**Q1.** What are the most influential technologies of Industry 4.0 for creating more responsive and resilient supply chains in case of emergencies, such as the COVID-19 outbreak?

**Q2.** How can the technologies of Industry 4.0 enable supply chains to handle the effects of the COVID-19 outbreak and enhance the responsiveness of the supply chains?

## Methods

The study employs systemic literature review (SLR) methodology to get a thorough insight into the relevance of Industry 4.0 technologies in the supply chain during COVID-19.

Researchers have recommended SLR as a comprehensive literature review framework. ^72^ An overview of the SLR process ^73^ followed in this study is shown in Table 1.

**Table 1.** Summary of the systematic literature review.

**Phase 1**	**Research question**
Formulating of the research questions	Q1. What are the most influential technologies of Industry 4.0 for creating more responsive and resilient supply chains in case of emergencies, such as the COVID-19 outbreak? Q2. How can the technologies of Industry 4.0 enable supply chains to handle the effects of the COVID-19 outbreak and enhance the responsiveness of the supply chains?
**Phase 2 & Phase 3**	**Electronic databases**
	Scopus ( scopus.com ), ProQuest ( proquest.com ), Google Scholar ( scholar.google.com )
	**Database setting** **Journal articles** **Book chapters** **Conference proceedings** **English language only**
**Search period**	2016-2021
**Method**	PRISMA
**Phase 4** Assessment of findings	**Analysis segment** Iterative compilation of the documents
**Phase 5** Reporting of findings	**Synthesis segment** Emerged perspective and results are extracted from documents and discussion

**Figure 1.**
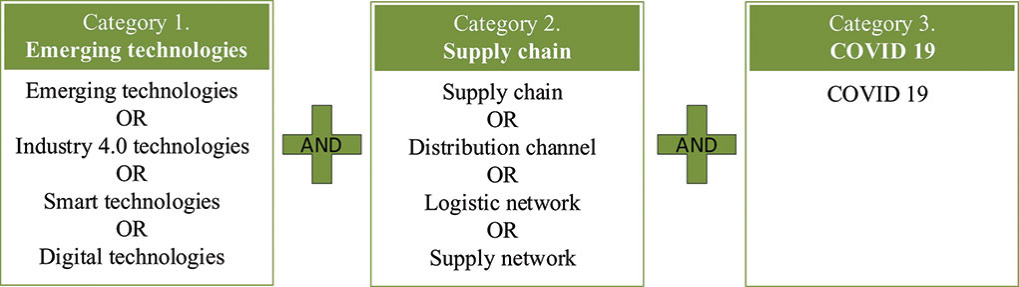
Categorical keywords for literature search.

**Figure 2.**
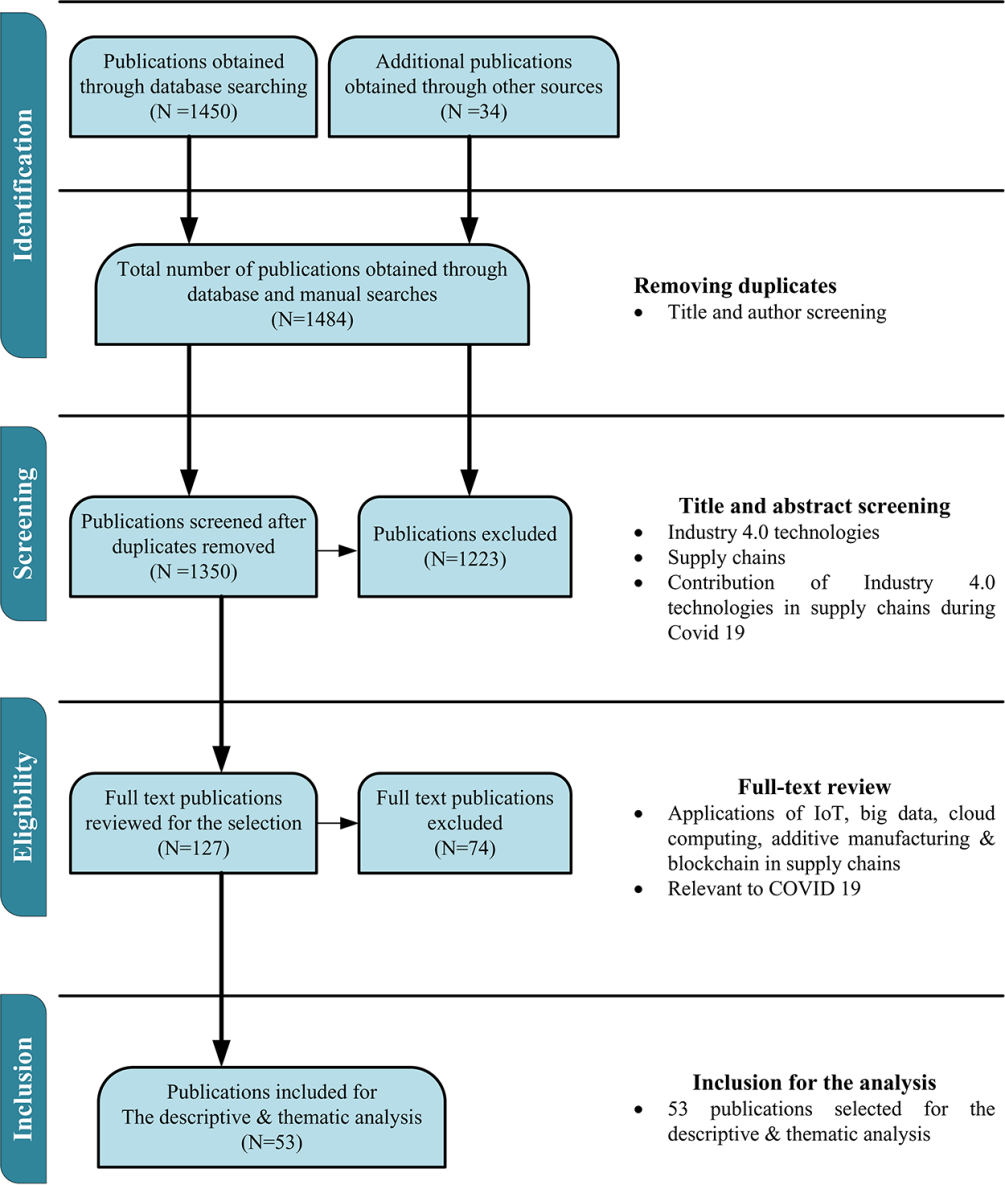
“Preferred Reporting Items for Systematic Review and Meta-analysis Protocols (PRISMA)”.

To create a repeatable and impartial search method, the researchers only referred to the most relevant publications connected to the topic. Figure 1 illustrates the three categorical keywords used by the authors to find the most relevant publications. The study adopted the “Preferred Reporting Items for Systematic Review and Meta-analysis Protocols (PRISMA)” framework developed by Moher ^74^ and the flowchart is visualized in Figure 2. The drafting process was utilised to extract the most relevant articles on the effect of COVID-19 on supply chains and the potential of the emerging technologies to resuscitate supply chains, as stated in the PRISMA standards.

## Results

Fifty-three articles were included for the descriptive and thematic analysis. The reviewed publications showed that the emerging technologies play a significant role in rescuing the disrupted supply chains during the COVID-19 pandemic. The following sub-sections illustrate the descriptive analysis of the publications.

### Type of publications

**Figure 3.**
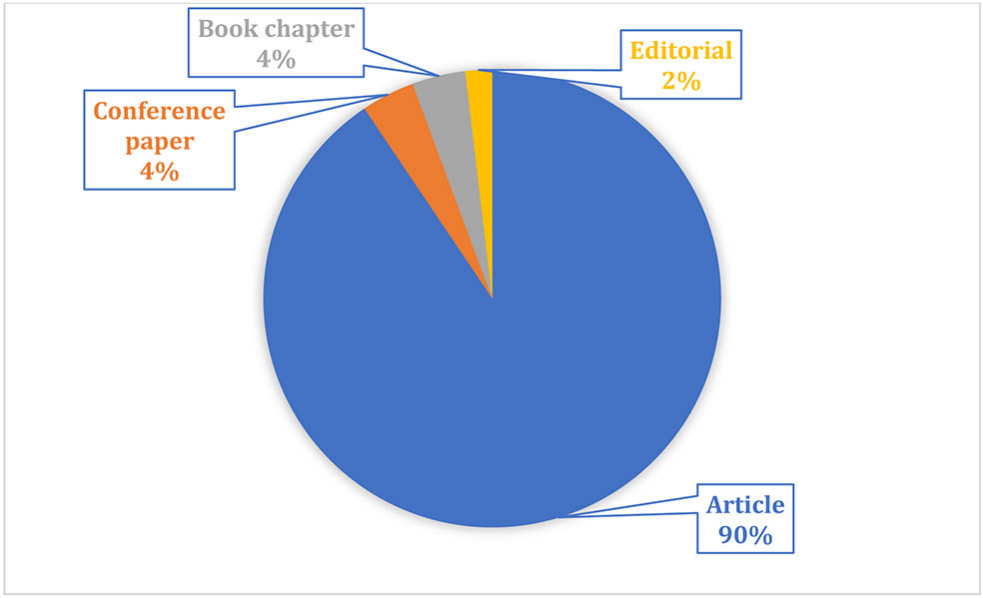
Types of publications.

The descriptive findings of 53 articles are shown in the frequency analysis. Figure 3 depicts a high-level representation of the results. Out of the 53 papers, 48 journals provide 90% of the articles, two conference papers and two book chapters account for 4% each of the total publications, and 2% are the culminating articles.

### Year-wise distribution of the publications

**Figure 4.**
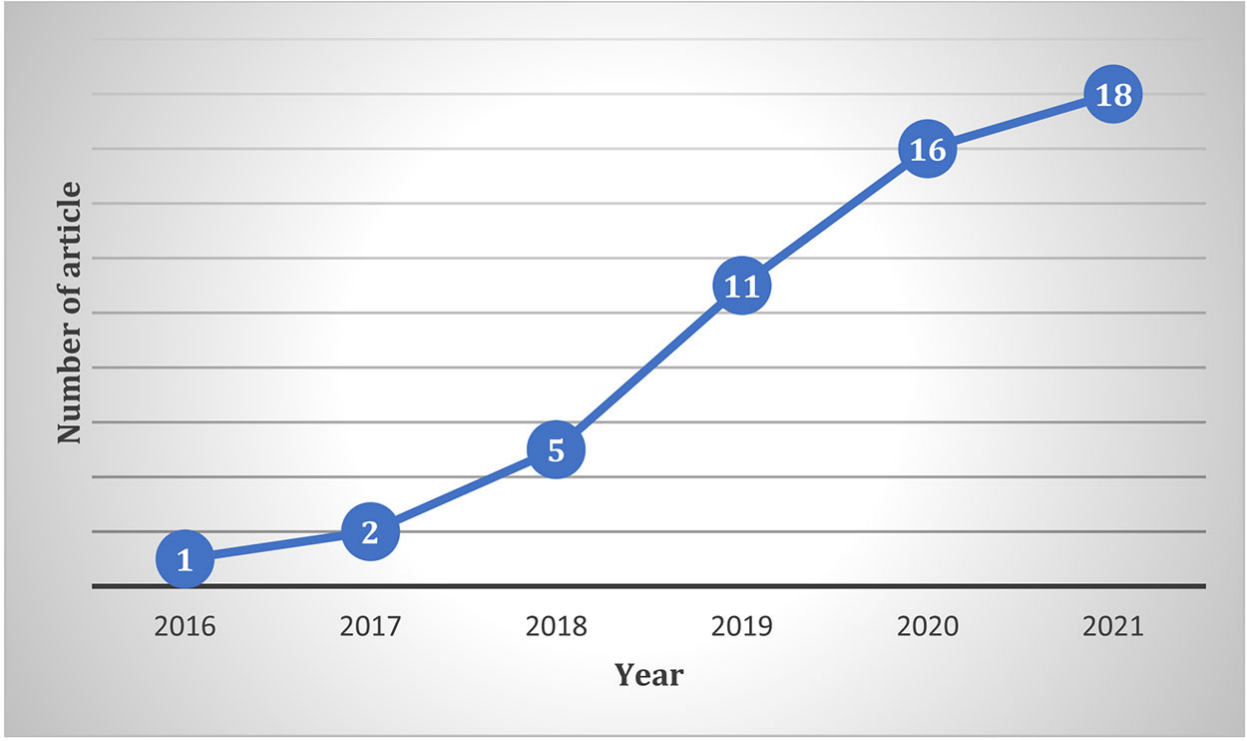
Year-wise distribution of the publications.

The year-wise distribution of the articles is shown in Figure 4. The publication trend demonstrates an impressive growth in the literature, indicating that the topic is well recognised among academics.

### Journal-wise distribution of the publications

**Figure 5.**
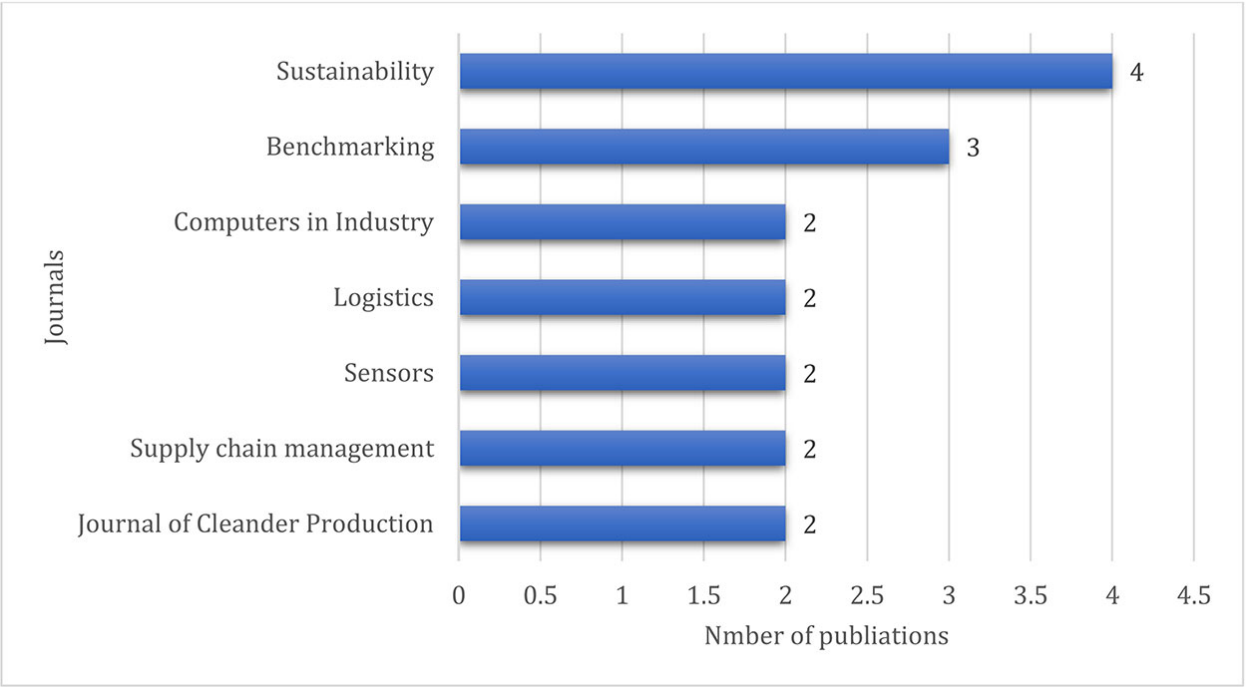
Journal-wise distribution of the publications.

Figure 5 presents the distribution of publications among the top seven journals. Sustainability (MDPI) tops with four articles, and benchmarking followed with three articles.

### Contribution of the publishers

**Figure 6.**
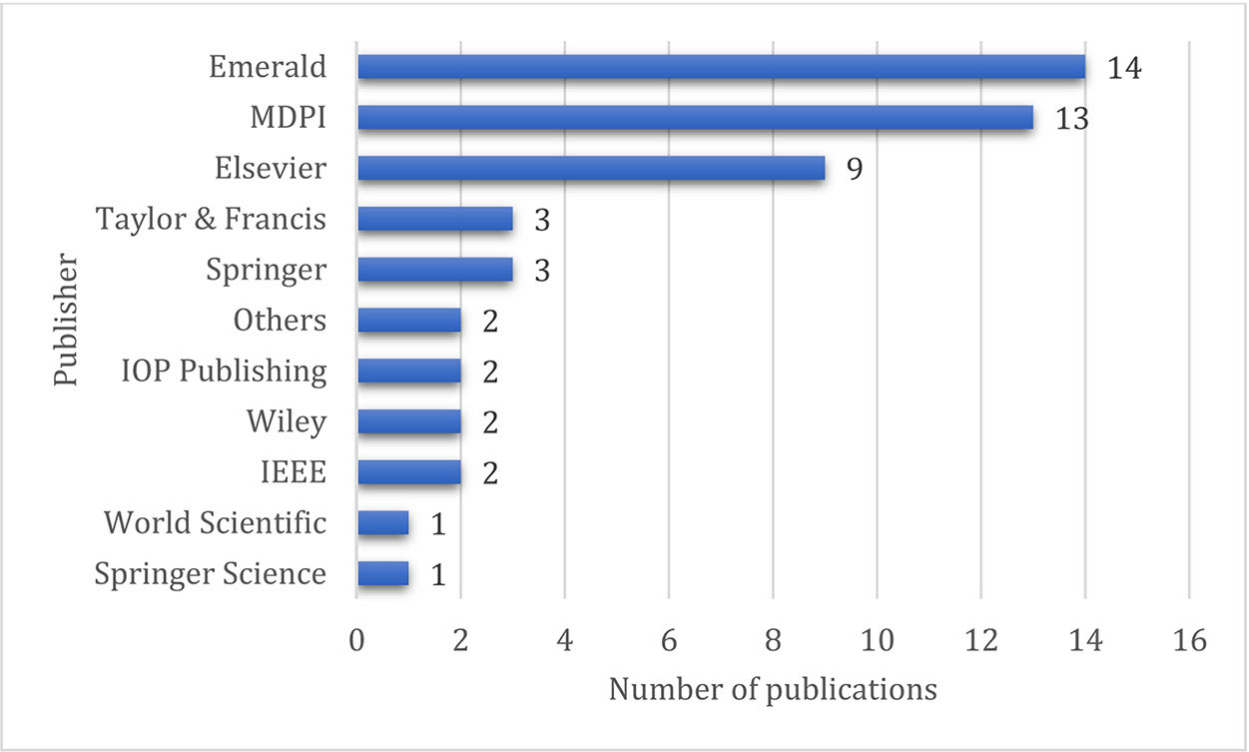
Contributing publisher.

The contributions of different publishers are shown in Figure 6. Emerald has the most publications with fourteen papers, followed by MDPI and Elsevier with thirteen and nine articles, respectively. This indicates that the concept of Industry 4.0 and supply chains is widely covered.

### Research method-wise distribution

**Figure 7.**
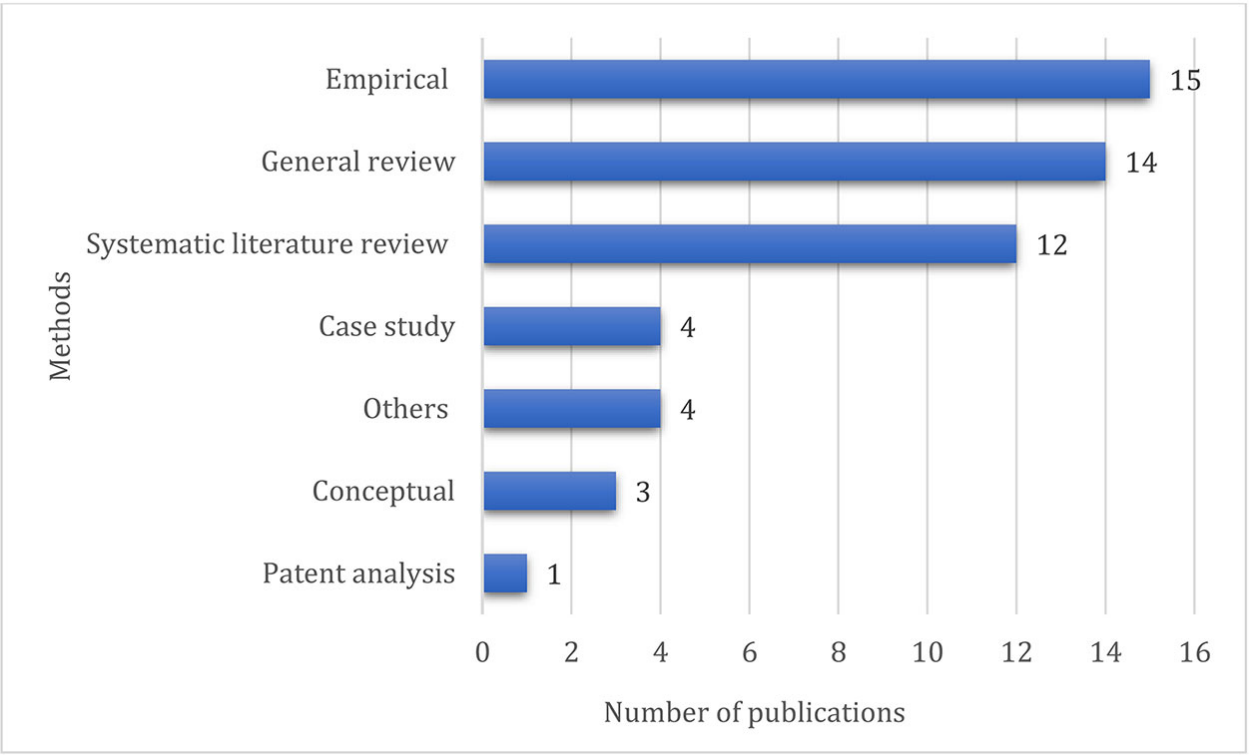
Research method-wise distribution.

The research method-wise distribution of the chosen 53 articles is shown in Figure 7. Empirical methodology tops with 15 papers, followed by general reviews and systematic literature reviews comprising 14 and 12 articles, respectively.

### Top cited publications

**Figure 8.**
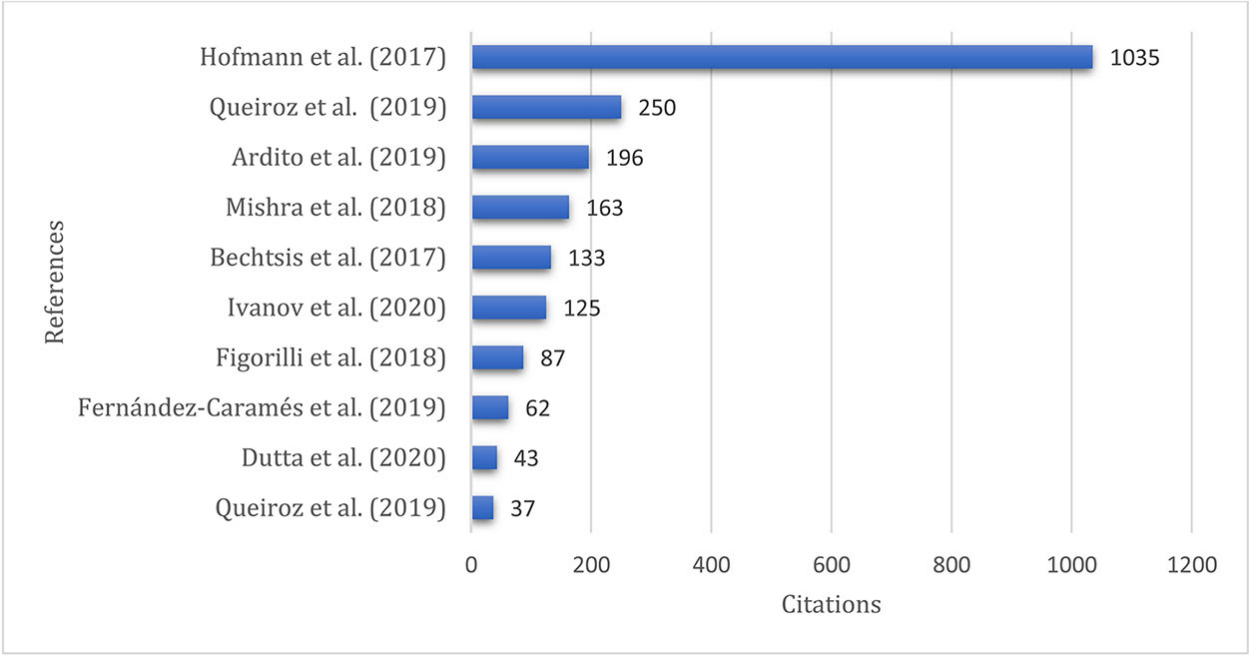
Top cited publications.

Figure 8 demonstrates the top-cited articles, and the publication by *Hofmann, Sternberg, Chen, Pflaum and Prockl* ^24^ leads the list with 1035 citations, followed by *Queiroz and Wamba* ^44^ and *Ardito, Petruzzelli, Panniello and Garavelli* ^4^ with the second and third highest number of citations, 250 and 196, respectively.

### High-contributing authors

**Figure 9.**
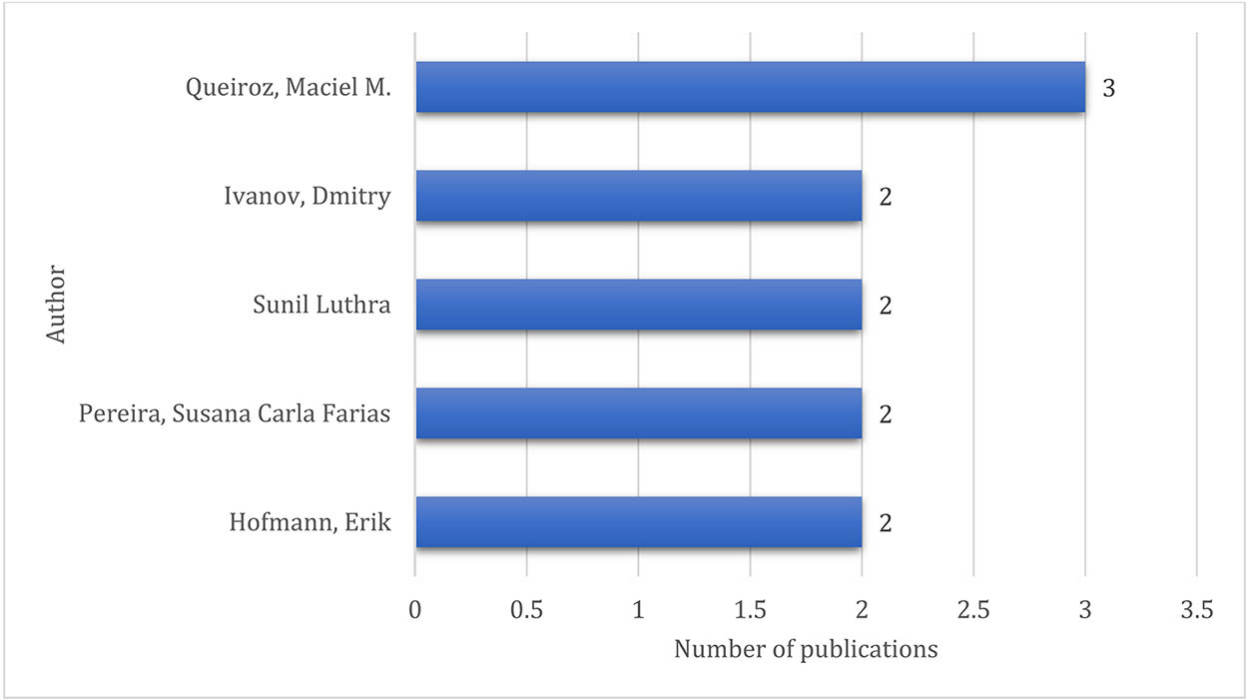
High contributing authors.

The list of high-contributing authors is shown in Figure 9. Maciel M. Queiroz tops the list with three publications, followed by Hofmann, Erik; Pereira, Susana Carla Farias; and Sunil, Luthra and Ivanov, Dmitry with two publications each.

### Country-wise publications

**Figure 10.**
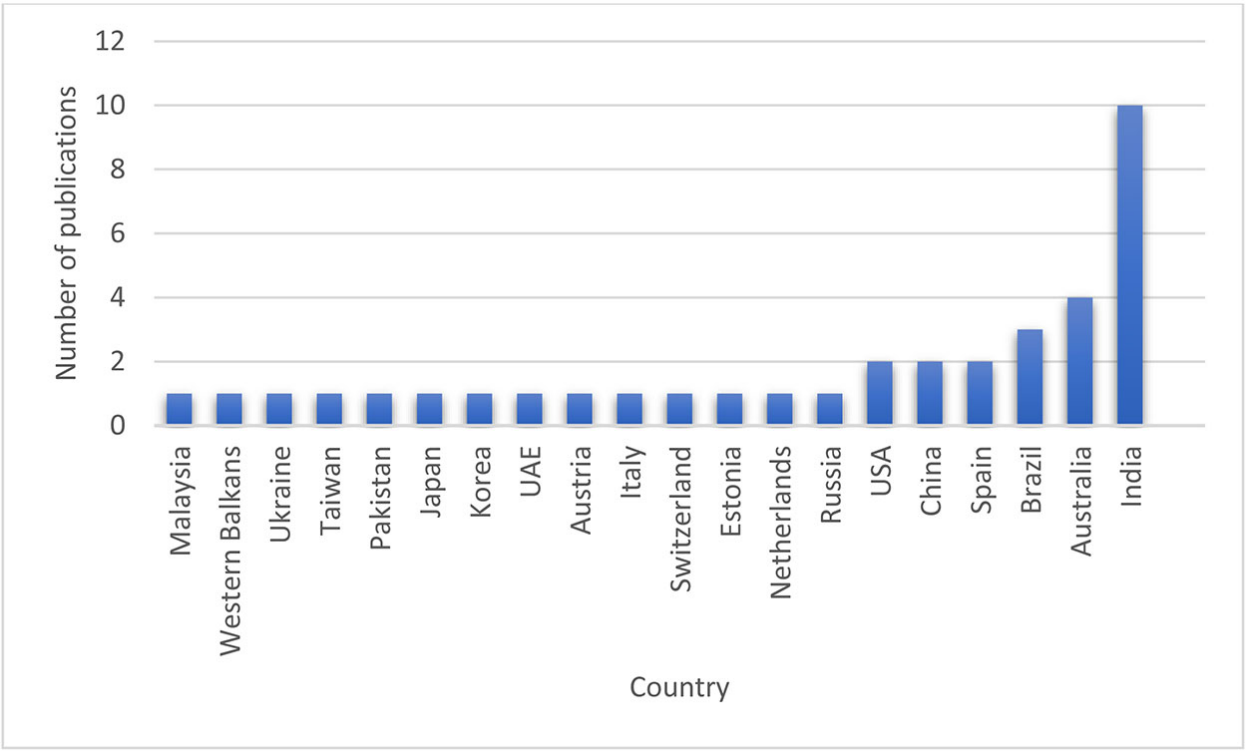
Country-wise distribution.

Figure 10 shows that India tops the list with ten articles, followed by Australia and Brazil with four and three papers, respectively, then the USA, China and Spain, each with two articles.

### Sector-wise distribution

**Figure 11.**
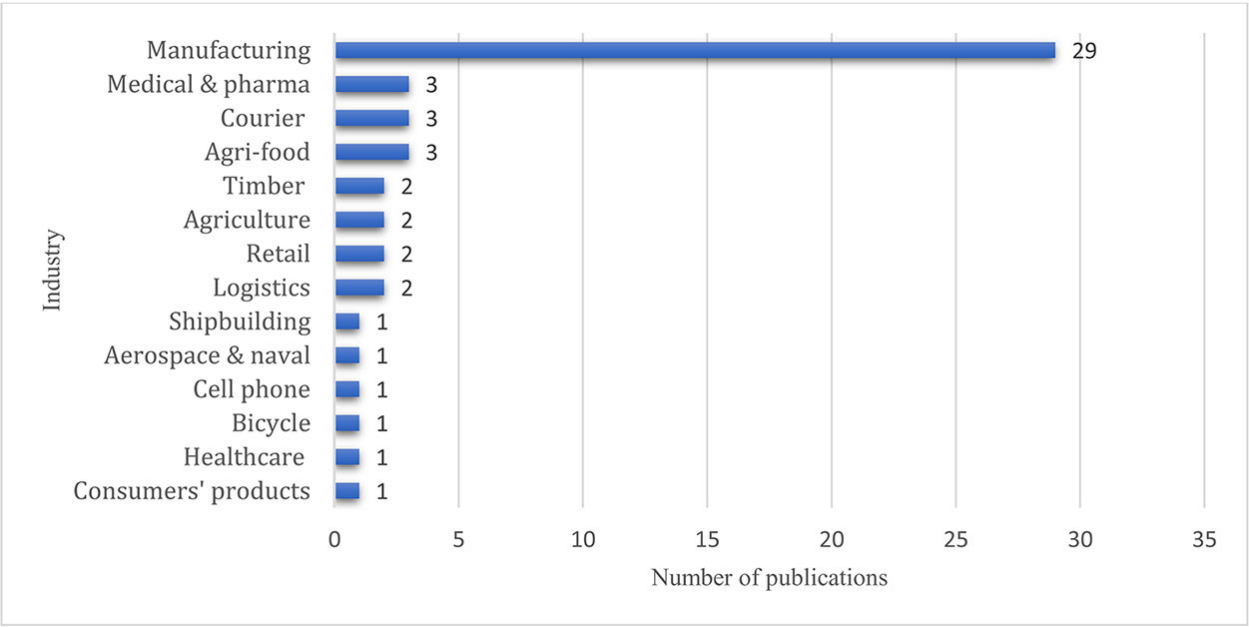
Sector-wise distribution

Figure 11 illustrates the sector-wise distribution, and the manufacturing supply chains accounts for 29 articles out of 53. Medical & pharmaceuticals, courier, and agri-food supply chains are reflected in each of the three articles.

## Discussion

Supply chains have been significantly disrupted during COVID-19, and the fundamental concerns addressed primarily are demand instability and supply shocks. ^57, 58, 61, 63, 64, 75– 80^ COVID-19 not only created havoc in demand and supply but also altered the spending patterns of consumers ^80, 82^ and led to inflation. ^76, 79, 83^ The pandemic also caused reduced sales ^75, 76, 84, 85^ and business shutdowns ^61, 78, 84^ with huge economic losses in various industries like cars, tourism, and transportation. ^58, 82^ This resulted in a shortage of workers ^78, 85^ and raw materials ^63, 64, 78^ and a simultaneous massive failure of production capacity. ^75, 84^ This section discusses the recovery strategy for the disrupted supply chains during the COVID-19 pandemic by deploying emerging technologies. The role of these technologies in reviving the supply chains is also underlined.

### Recovery strategy through the employment of the emerging technologies

With Industry 4.0 technologies, the automated and digital supply chain can be the best solutions for recovering the disrupted supply chains during the COVID-19 pandemic. The role and function of disruptive technologies are essential. ^3– 5^

The literature review highlights the emerging technologies that may streamline supply chain resilience, resulting in increased robustness during an emergency or an unexpected and dynamic catastrophe. The results are summarized in Table 2.

**Table 2.** The emerging technologies in various supply chains.

Technologies	References
Internet of Things	1- 36
Big data	1, 3- 6, 8- 11, 14- 19, 21- 25, 27- 29, 31- 36, 38- 42
Cloud computing	1, 3, 4, 6, 9, 10, 12, 13, 16- 20, 22- 29, 32- 35, 39, 40, 45, 46
Additive manufacturing	1, 3, 4, 8, 9, 16, 17, 22- 24, 28, 29, 32- 35, 39, 45, 47
Blockchain	1, 3, 12, 13, 16, 17, 19- 21, 24, 27- 29, 31, 33- 35, 39, 40, 42

The manufacturers and producers may adopt and execute emerging technologies such as the Internet of Things (IoT), big data, cloud computing, additive manufacturing, and blockchain to continue, rescue, and recover their supply chains that are affected by the pandemic. Management should dedicate more effort to an automated supply chain developed by the emerging technologies.

## The role of the emerging technologies in supply chains

COVID-19 emphasises the whole production process and the structural factors that link Industry 4.0 technology, supply chains, and the COVID-19 pandemic. In this section, these factors are examined based on the reviews. Table 3 illustrates the role of the emerging technologies in reviving supply chains that favour long-term supply chain performance. The key approaches of the supply chains include real-time information and transparency to manage customer demand effectively, ^1, 4^ improved interaction with suppliers and vendors, ^10, 52^ and optimising the supply chain to satisfy the needs of the company. ^40, 53^ A supply network integrated with emerging technologies enables companies to build faster, flexible, accurate, and efficient supply chains. ^7^ These approaches have a significant influence on supply chain resilience, leading to increased robustness in the face of an emergency or abrupt and large-scale calamities. ^22^ These technologies will improve industrial processes across the horizontal value chain, including engineering, material utilisation, supply chain, and product life cycle management, ^33, 60, 86^ along with opportunities such as improvements in operations, energy conservation, and logistic support. ^17, 19, 87^ Productive competency, ^23, 48^ waste reduction, ^1, 17^ inventory management, ^19, 25^ information sharing with supply chain members, ^54, 55^ tracking and tracing warehouse inventory, ^29, 88^ and logistics information ^60^ are guaranteed by Industry 4.0 technologies. ^1, 21, 22^ Via data sharing, these technologies facilitate a decrease in local and international bulk cargo transit, delivery mistakes, and needless waiting periods, as well as the prevention of products being damaged. ^23, 48^ The review indicates that Industry 4.0 technologies contribute to improving operations management ^4, 5, 7^ and manufacturing processes creating customized products. ^89^

**Table 3.** The role of the emerging technologies in various supply chains.

Category	Advantages	Sources
Monitoring	Customers’ demand management	1, 3- 5, 8, 35, 37
	Supply management	36, 43, 44
	Sales and operations planning	16, 22, 39
	Product portfolio management	30, 48
	Risk management	17, 34, 44, 49
Integration	Integration across the inter-intra organisation boundaries	25, 50
	Information sharing among the supply chain members	51, 52
	Supplier and customer integration	10, 14, 17, 48
Responsiveness	Quick response to customer demand	4, 14, 40, 53
	Delivery in a timely manner	1, 14, 32, 54
Information sharing	Accessing real-time data by different members of the supply chain	12, 14, 15, 55
	Tracking logistics information	27, 59, 60
	Tracking and tracing warehousing information	29, 33, 61
Inventory management	Obtain a holistic view of inventory levels	19, 29, 62
Waste reduction	Real-time information sharing enables in waste reduction	1, 13, 17, 41, 55
Production efficiency	Utilization of all resources enables production efficiency	23, 26, 48

## Limitations and future studies

The authors note several limitations in the study. First, the findings are derived considering English language-publications only, and those written in other languages are excluded. Future research may provide additional insights by reviewing the literature written in other languages. Second, the authors focus on the literature only in the context of Industry 4.0. Thus, the holistic view of Industry 4.0 has not been evaluated in this study. Furthermore, the study reviewed the role of the five major technologies such as IoT, big data, cloud computing, additive manufacturing, and blockchain and discussed how these technologies could be employed to revive the supply chains during emergencies. Future studies may include other emerging technologies such as artificial intelligence, robotics, augmented reality, and simulation/digital twins to get a broader range of findings. In spite of these constraints, the current study adds to the identification of significant technologies and their roles in supply chain management in the area of Industry 4.0.

## Conclusion

Recent studies have emphasized the impact of individual technologies on the supply chain, such as IoT, big data, cloud computing, additive manufacturing, and blockchain, and how these technologies support companies in achieving competitive advantage. However, comparatively few studies have explored the influence of these technologies concurrently, particularly during an unexpected situation. The present study is based on these gaps and responds to the research questions using a systematic literature review. In answering the first research question, the study confirmed that most publications highlight IoT, big data, cloud computing, additive manufacturing, and blockchain ( Table 2) that may assist in building resilient and robust supply chains, even in the COVID-19 era. Regarding the second research question, the literature indicates that the roles and functions ( Table 3) played by these technologies lead to establishing integrated, flexible, robust, responsive, efficient, and competent supply chains. The study also reveals unexplored features of supply chains. Therefore, highlighting a discussion on implementing Industry 4.0 technologies in supply chain studies offers an interesting future research topic.

## Data availability

### Underlying data

All data underlying the results are available as part of the article and no additional source data are required.

### Reporting guidelines

Figshare: PRISMA Checklist_The Implications of Industry 4.0 on Supply Chains Amid the Covid 19 Pandemic – A Systematic Literature Re.docx, https://doi.org/10.6084/m9.figshare.16602356.

Data are available under the terms of the Creative Commons Attribution 4.0 International license (CC-BY 4.0).
