# The implications of Industry 4.0 on supply chains amid the COVID-19 pandemic: a systematic review

**DOI:** 10.12688/f1000research.73138.2

**Published:** 2022-01-31

**Authors:** Mohammad Nurul Hassan Reza, Sreenivasan Jayashree, Chinnasamy Agamudai Nambi Malarvizhi, Md Abdur Rauf, Kalaivani Jayaraman, Syed Hussain Shareef

**Affiliations:** 1 Faculty of Management, Multimedia University, Cyberjaya, Selangor, 63100, Malaysia; 2 Faculty of Educational Study, University Putra Malaysia, Serdang, Malaysia, 43400, Malaysia; 3 Faculty of Accountancy and Business, Universiti Tunku Abdul Rahman, Bandar Sungai Long, Selangor, 43000, Malaysia; 4 NTT DATA Business Solutions, Cyberjaya, Selangor, 63000, Malaysia

**Keywords:** Industry 4.0, emerging technologies, supply chain, COVID 19, Systematic Literature Review

## Abstract

**Background**: COVID-19 has caused significant disruptions in supply chains. It has increased the demand for products and decreased the supply of raw materials. This has interrupted many production processes. The emerging technologies of Industry 4.0 have the potential to streamline supply chains by improving time-sensitive customized solutions during this emergency.

**Purpose**: The study identifies the core technologies of Industry 4.0 and the role and impact of these technologies in managing the disruption caused by the COVID-19 outbreak in strengthening the supply chain resilience.

**Design/methodology/approach:** An extensive literature review using the “Preferred Reporting Items for Systematic Review and Meta-Analysis” method was carried out on the impact of the COVID-19 pandemic on supply chains and Industry 4.0 technologies. The study was undertaken by selecting keywords validated by experts, and a search was conducted in the Scopus, ProQuest, and Google Scholar databases. Publications from the leading journals on these topics were selected. The bibliographical search resulted in 1484 articles, followed by multiple layers of filtering. Finally, the most pertinent articles were selected for review, and a total of 42 articles were analyzed.

**Findings:** The findings of the study showed that the majority of the articles emphasized the digitalization of supply chain management, acknowledging the fundamentals, applications, and prospects, revealing the drivers and challenges of Industry 4.0 technologies to manage disruptions. Most of the authors identified IoT, big data, cloud computing, additive manufacturing, and blockchain to maintain the supply chain resilience.

**Originality/value:** Existing literature on epidemics lacks the basics and practices of utilizing Industry 4.0 technologies in the supply chain recovery process. To fill this research gap, the study summarizes the potential of Industry 4.0 technologies to lessen supply chain disruptions caused by COVID-19. The study findings are valuable for policymakers and practitioners and contribute to supply chain management studies.

## Introduction

The COVID-19 pandemic has already had a crucial impact on human health as well as countries’ economies. Supply chains in various industries have been under tremendous pressure to avoid considerable disruptions in their operations. ^1^ COVID-19 has also affected every member of the supply chain process. ^2^^,^^3^ The failure of many nations and businesses to deal with the COVID-19 pandemic is attributable to their supply chains and their inability to deliver products and services. ^4^ Supply chain issues, those associated with sourcing techniques, have created substantial disruptions in various supply chains. Lack of risk management, adoption of the single-sourcing strategy, and supplier delivery delays are examples. ^5^ These distractions have generated numerous lessons in understanding supply chain management, advising both researchers and practitioners to reconsider how supply chain strategies should address new disruptive threats. ^1^ In this regard, incorporating Industry 4.0 technologies has become a strategic imperative for supply chains to improve their competitiveness in the volatile, dynamic or even in any crisis. ^6^^,^^7^ Industry 4.0 fosters decentralized manufacturing systems, ^8^ offers a business atmosphere integrating humans, machines, equipment, and operational processes through Cyber-Physical Systems and the Internet. ^9^ Industry 4.0 integrates its emerging technologies into the entire organizational setting, ^10^ facilitates automated and dynamic production systems, ^10^^,^^11^ significantly improves the quality of products and services by digitizing the operational activities. ^12^

Existing studies revealed that Industry 4.0 technologies, such as the Internet of Things, ^13^^–^^15^ big data, ^16^^–^^18^ cloud computing, ^19^^,^^20^ additive manufacturing, ^21^ and blockchain, ^22^^–^^24^ would enable firms to remotely monitor, operate and inspect supply chain activities, emphasized by COVID-19. These technologies have shown to be a significant facilitator, with superior speed and scalability compared to human labor, allowing organizations to manage the disruption caused by the COVID-19 pandemic. ^6^ The integration with cutting-edge technologies assists in establishing digital supply chains through automated and self-executed procedures, ^1^ essential to maintain supply chain resilience during any crisis. Product identification, location, and other tracking information are critical for firms to keep track of their products at all supply chain stages in this disrupted situation. ^25^ Using Big Data analytics, supply chain managers may gain a holistic insight in recognizing the customer demand and maximizing the efficiency of their organization for supply chain management. ^17^^,^^26^ Cloud-based data collection and analysis for demand management are effective in sudden demand changes. ^27^ Using sensors, tags, actuators, and other IoT-based devices enables establishing a complete cyber-physical supply chain. ^15^^,^^26^^,^^28^ By utilizing real-time data, the emerging technologies allow suppliers to respond quickly and transparently to any demand changes. ^19^ The real-time data also enable firms to respond rapidly to customer orders or even automatically receive orders from the cloud-based platform to produce goods. ^1^ However, additive manufacturing may be explored to boost manufacturing flexibility by mass customization, enhancing supply chain performance and competencies. ^29^ The technologies would be used to convey the customer demand directly to manufacturing or distribution hubs and create self-executed handling procedures at distribution hubs. ^30^ Thus, these technologies enable supply chain partners to deliver the product promptly or shorten the delivery time. The IoT, big data, and cloud computing would allow a digital warehouse management system to provide real-time visibility of all the products and warehouse functions, enhancing proficiency and eliminating inventory shortages. ^31^ These technologies also enable organizations to track upstream supply chain processes, allowing better inventory management during disruptions. ^13^^,^^30^

However, the COVID-19 pandemic emerged when the supply chains had been called upon to transform and adapt the dynamics of Industry 4.0. Yet, the existing literature on the fundamentals of Industry 4.0 concentrates more on the utilization of different technologies, such as IoT, big data, cloud computing, and additive manufacturing in the context of manufacturing, ^14^ rather than integrating supply chain management techniques. ^32^ As the concept of Industry 4.0 and it’s related technologies are recently developed, previous studies on epidemics rarely address the employment of emerging technologies in the recovery process ^33^ as well as the impact on commercial supply chains. ^34^ Consequently, the employment of these technologies in supply chains, relevant tools, techniques and applications remain unclear, ^2^ which requires a holistic approach. ^35^ Furthermore, existing literature lacks a comprehensive review on the role of new technologies in enabling supply chains, especially in emergencies such as the COVID-19 pandemic. ^1^^,^^2^ Although some studies addressed the issue from a narrow perspective. For example, Gupta ^36^ demonstrated the potential of big data analytics in enhancing supply chain visibility, Amer ^37^ revealed the prospects of IoT in the food supply chain. Brandtner ^17^ illustrated the role of digital supply chain in improving sales and operations planning strategies. Focusing on the food supply chain, Galanakis ^18^ examined the prospects of Industry 4.0 technologies. P. Dutta ^22^ summarized the key benefits of blockchain in supply chains. Therefore, Chowdhury, Paul, Kaisar and Moktadir ^2^ and Frederico ^1^ suggested looking into the role of emerging technologies of Industry 4.0 in regulating the effects of COVID-19. The current study has conducted a systematic literature review to close this gap. Also, the study assesses the overall role of Industry 4.0 technologies in developing a holistic supply chain framework and focuses on the potential applications of the emerging technologies to address pandemic-related supply chain problems.

Hence a comprehensive literature review was conducted on Industry 4.0 technologies, supply chain, and COVID-19 for exploratory analysis and a deeper understanding to answer the following research queries:

**Q1.** What are the most influential technologies of Industry 4.0 for creating more responsive and resilient supply chains in case of emergencies, such as the COVID-19 outbreak?

**Q2.** How can the technologies of Industry 4.0 enable supply chains to handle the effects of the COVID-19 outbreak and enhance the responsiveness of the supply chains?

The remainder of the study illustrates the main sections, including methods, results, discussion, limitation, and conclusion. The methodology employed for the study is discussed in methods, descriptive analysis, and summary of the reviewed literature are demonstrated in the results. The discussion section is presented with the categorical analysis and research gaps, followed by the limitations and recommendations for future study. Finally, the study concludes with a brief discussion answering the research questions.

## Methods

The study employs systemic literature review (SLR) methodology to get a thorough insight into the relevance of Industry 4.0 technologies in the supply chain during COVID-19.

Researchers have recommended SLR as a comprehensive literature review framework. ^38^ An overview of the SLR process ^39^ followed in this study is shown in Table 1.

**Table 1.** Summary of the systematic literature review.

**Phase 1**	**Research question**
Research question formulation	Q1. What are the most influential technologies of Industry 4.0 for creating more responsive and resilient supply chains in case of emergencies, such as the COVID-19 outbreak? Q2. How can the technologies of Industry 4.0 enable supply chains to handle the effects of the COVID-19 outbreak and enhance the responsiveness of the supply chains?
**Phase 2**	**Electronic databases**
	Scopus ( scopus.com), ProQuest ( proquest.com), Google Scholar ( scholar.google.com)
	**Database setting** Journal articles Book chapters Conference proceedings English language only
**Phase 3**	Keyword search
**Search period**	2019-2021
**Phase 4 Inclusion/exclusion criteria**	**PRISMA** Identification Screening Eligibility checking Final selection
**Phase 5 Results**	**Descriptive analysis** Iterative compilation of the documents
**Phase 6 Discussion**	**Categorical analysis** Emerged perspective and results are extracted from documents and discussion

### Phase 1. Research questions formulation

Considering the supply chain disruptions caused by the COVID-19 and the importance of establishing digital supply, the study aims to scrutinize, in a systematic way, the most contributing technologies of Industry 4.0 to recover disrupted supply chains. In particular, the study addresses and developed two research questions ( Table 1). Accordingly, the research goal is to identify the most influential technologies of Industry 4.0 and their role in assisting organizations in revitalizing the supply chains disrupted by the pandemic.

**Figure 1.**
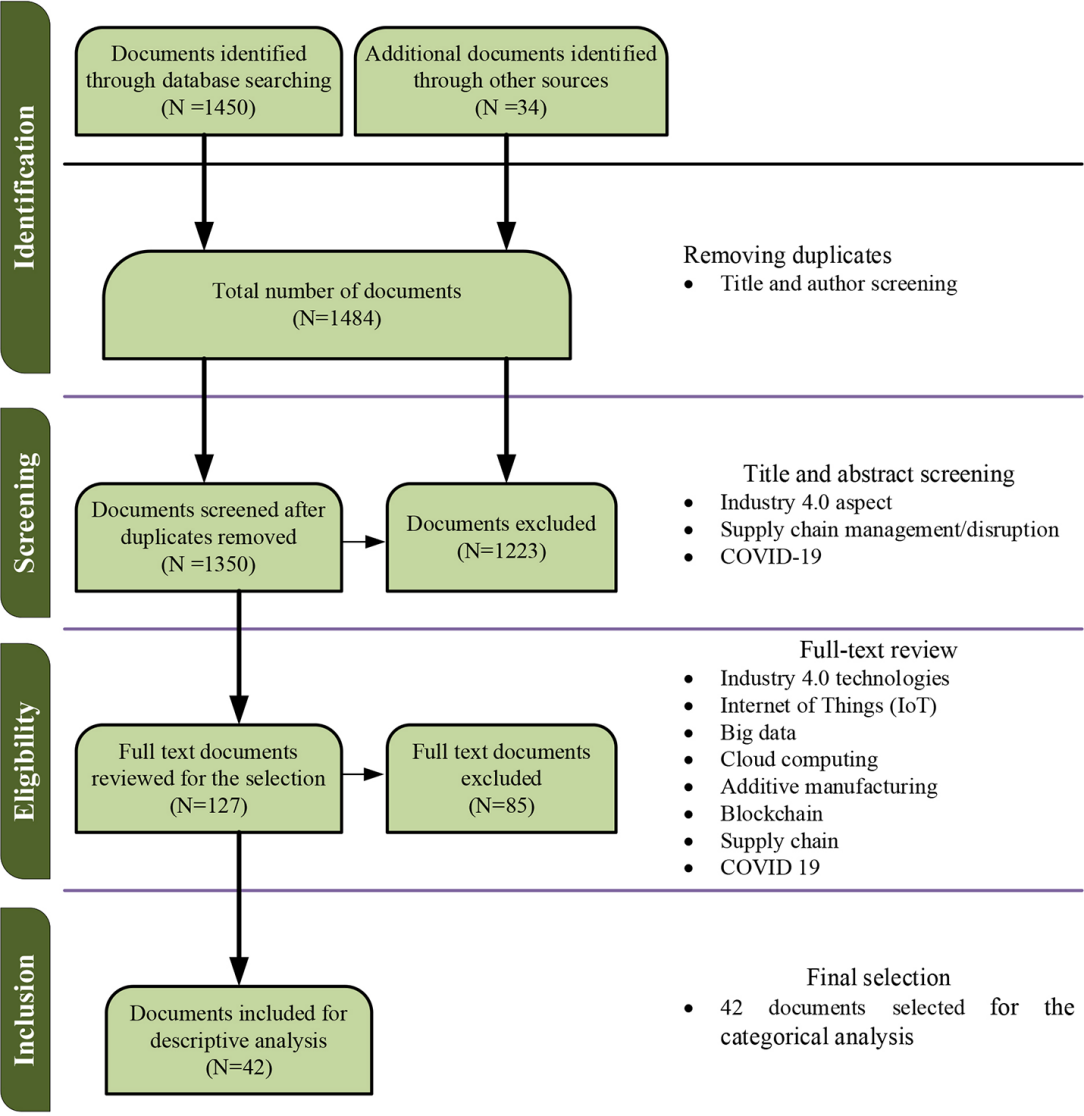
Preferred Reporting Items for Systematic Review and Meta-analysis Protocols (PRISMA).

To create a repeatable and impartial search method, the researchers only referred to the most relevant publications connected to the topic. The study adopted the “Preferred Reporting Items for Systematic Review and Meta-analysis Protocols (PRISMA)” framework developed by Moher ^40^ and the flowchart is visualized in Figure 1. The drafting process was utilised to extract the most relevant articles on the effect of COVID-19 on supply chains and the potential of the emerging technologies to resuscitate supply chains, as stated in the PRISMA standards.

### Phase 2. Database selection

The authors selected the main electronic databases, including Scopus, ProQuest, and Google Scholar, to find the most relevant documents for the study. The papers that fit the scope of the study have been chosen. As the COVID-19 emerged in 2019, the articles published from 2019 to 2021 were selected, which is under the area of the research.

### Phase 3. Keywords selection

**Figure 2.**
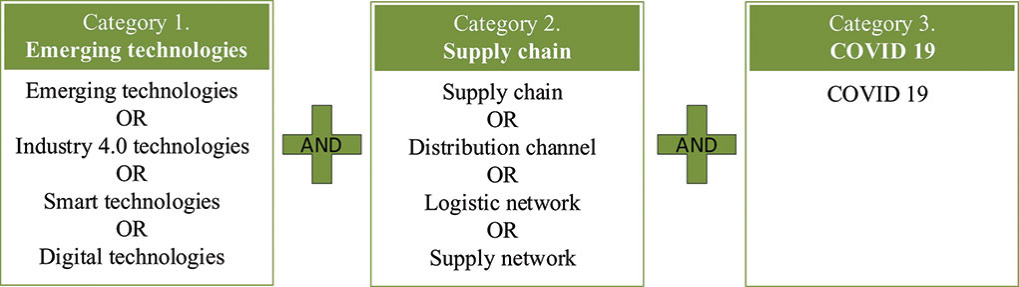
Categorical keywords for literature search.

The keywords used by the authors fall into three categories, as illustrated in Figure 2. A pairwise search was undertaken in May 2021; one keyword from each category was considered at a time. The authors ensured that the three categories of this study, i.e., emerging technologies, supply chain, and COVID-19, were covered. The keyword search included journal articles, book chapters, and conference proceedings.

### Phase 4. Article selection process

According to the PRISMA framework, the study followed four steps in the article selection process. The steps include identifying the articles, screening, eligibility, and final inclusion. Figure 1 visualizes the article selection flowchart.

***Identification***

The initial search queries resulted in a total of 1450 publications. A manual search was also conducted and produced 34 publications relevant to the study. So, the total number of publications was 1484. The authors used Endnote software to eliminate the duplicates to improve the results further.

***Screening***

After removing the duplicates, the number of articles dropped to 1350. The authors conducted a careful title and abstract screening and removed 1223 articles due to the irrelevancy to the subject of the study. The total number of eligible articles was reduced to 127 following the removal of 1223 items.

***Eligibility***

In this stage, the full-text review was done, and a total of 85 articles were excluded. The authors retained the articles contributing to supply chain studies relating to Industry 4.0 technologies during COVID-19 or supply chain disruptions.

***Inclusion***

After excluding 85 articles, 42 papers were selected for the final review. The authors retained at least three articles focusing on general supply chain disruptions rather than COVID-19. The review process was utilized to extract the most relevant articles illustrating the effect of COVID-19 or other natural disturbances on supply chains and the potential of the emerging technologies to resuscitate supply chains. The following considerations and analysis are based on the finally obtained 42 papers relevant to the current study. The following section discusses the bibliographic analysis of the selected articles.

## Results

The following section demonstrates the descriptive analysis of the selected articles. The core technologies of Industry 4.0 for supply chain resilience during COVID-19 are also identified in this section.

### Phase 5. Descriptive analysis

Forty-two articles were included for the descriptive and categorical analysis. The publication trend shows an immense surge in the literature and confirms that the researchers widely acknowledge the topic. The following sub-sections illustrate the descriptive analysis of the publications.

#### Type of publications

**Figure 3.**
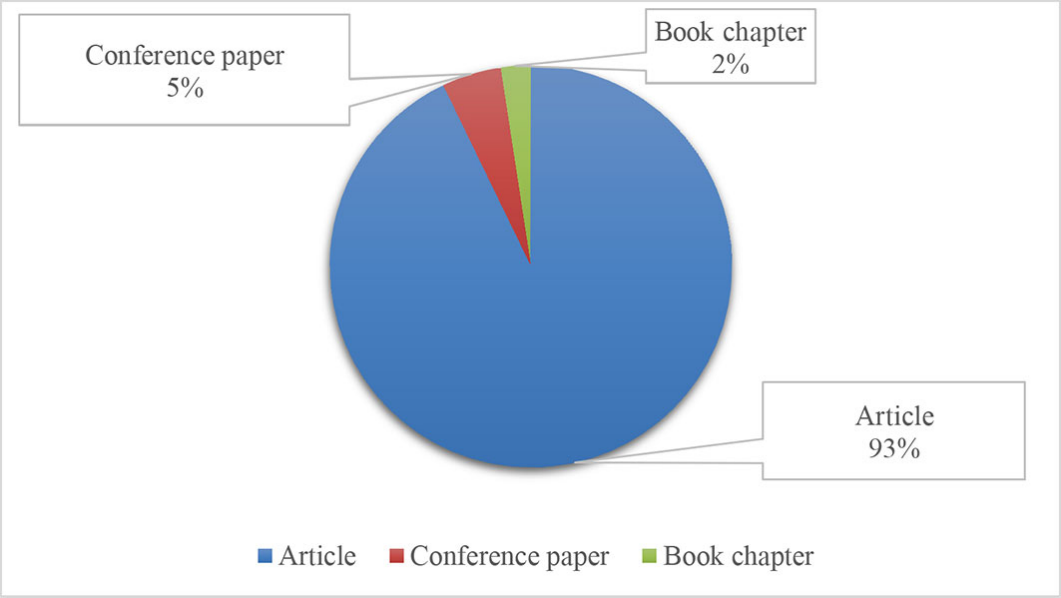
Types of publications.

The descriptive findings of 42 articles are shown in the frequency analysis. Figure 3 depicts a high-level representation of the results. Out of the 42 papers, 39 journals provide 93% of the articles, two conference papers and one book chapter account for 5% of the total publications, and 2% are the culminating articles, respectively.

##### Year-wise distribution of the publications

**Figure 4.**
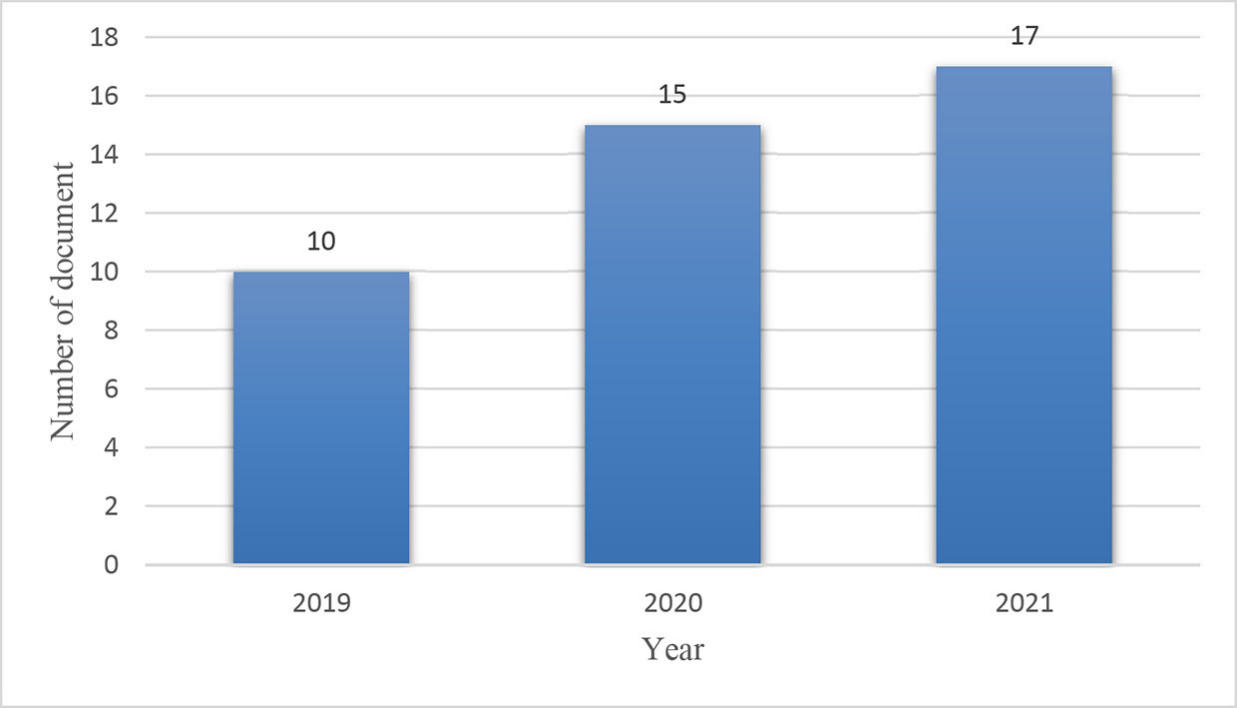
Year-wise distribution of the publications.

The year-wise distribution of the articles is shown in Figure 4. The figure illustrates the presence of a growing trend in the number of articles since 2019. Ten articles were published in 2019. A year later, in 2020, that number rose to 15, and as of May in 2021, it had reached 17. The publication trend demonstrates an impressive growth in the literature, indicating that the topic is well recognised among academics.

##### Journal-wise distribution of the publications

**Figure 5.**
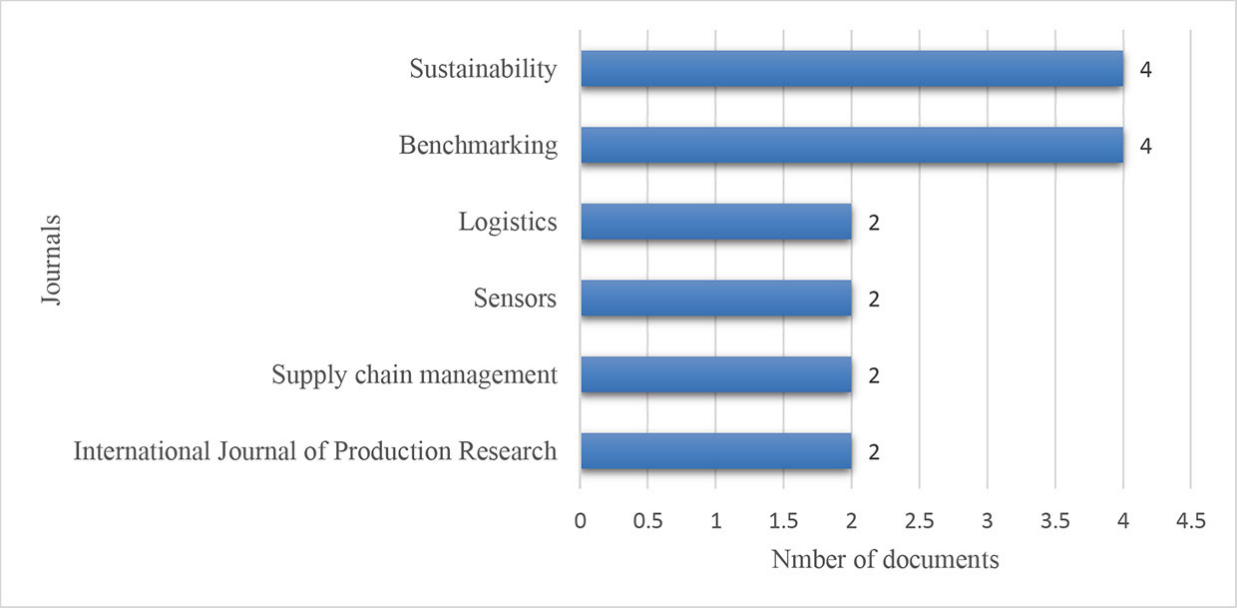
Journal-wise distribution of the publications.

A large number of journals have published papers on supply chain management relating to Industry 4.0 and COVID-19 disruption. Figure 5 presents the distribution of publications among the top six journals. Sustainability (MDPI) and Benchmarking topped with four articles, and Logistics, Sensors, Supply chain management, and International Journal of Production Research followed with two papers each.

***Contribution of the publishers***

**Figure 6.**
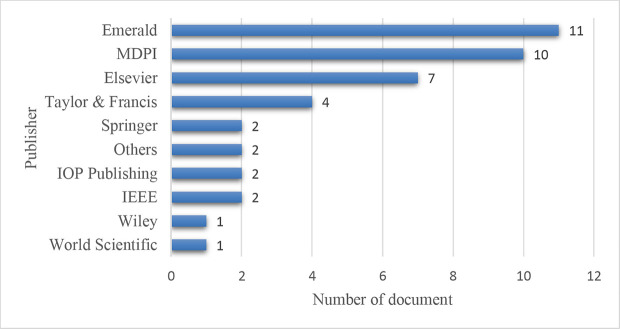
Contributing publisher.

The contribution of different publishers is shown in Figure 6. Emerald has the most publications with eleven papers, followed by MDPI with ten articles. Elsevier and Taylor & Francis stand with seven and four articles, respectively. Springer, IEEE, and IOP publishing produce two papers each. The volume of publications implies that the leading publishers have extensively covered industry 4.0 and supply chain management studies.

***Research method-wise distribution***

**Figure 7.**
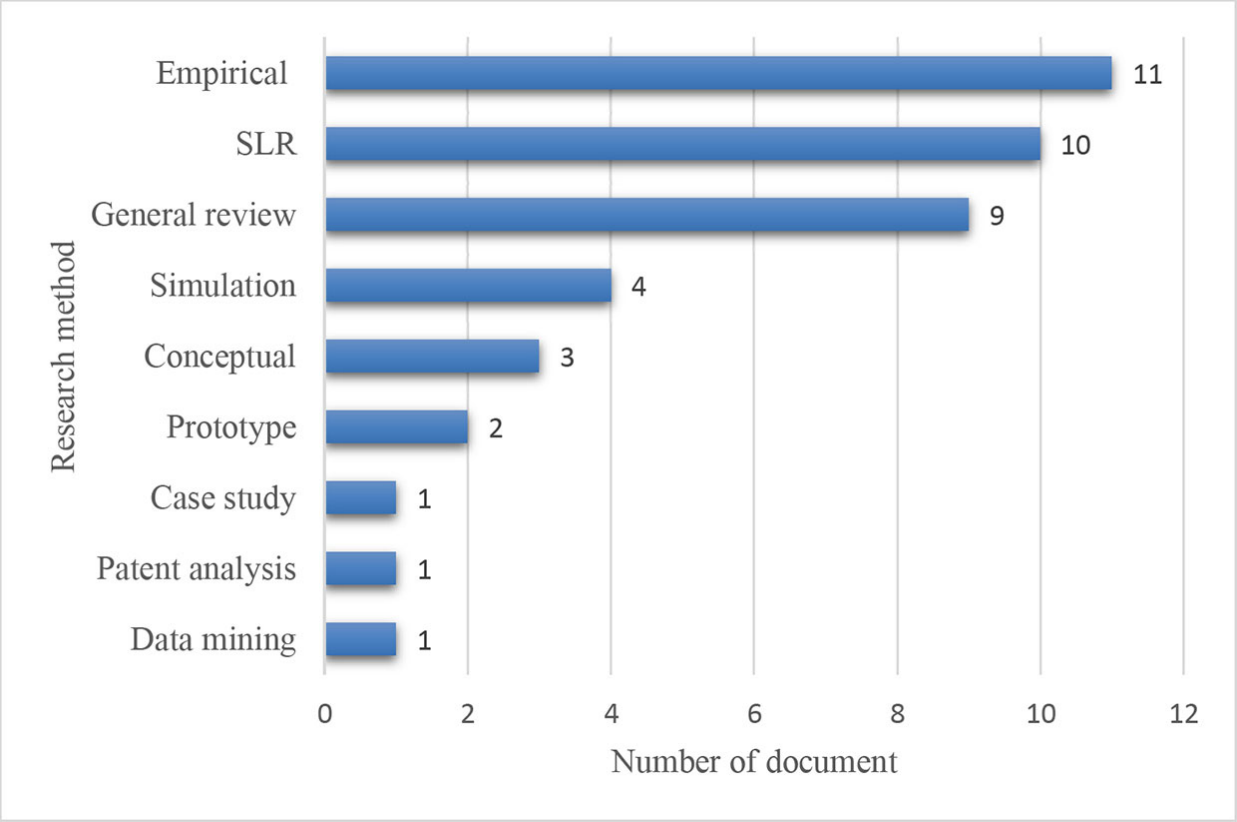
Research method-wise distribution.

The research method-wise distribution of the chosen 42 articles is shown in Figure 7. Empirical methodology topped with 11 papers, followed by systematic literature (SLR) and general reviews comprising 10 and 9 articles, respectively. 4 papers were found based on simulation. The number of conceptual and prototyping studies was 3 and 2, respectively.

***Top cited publications***

**Figure 8.**
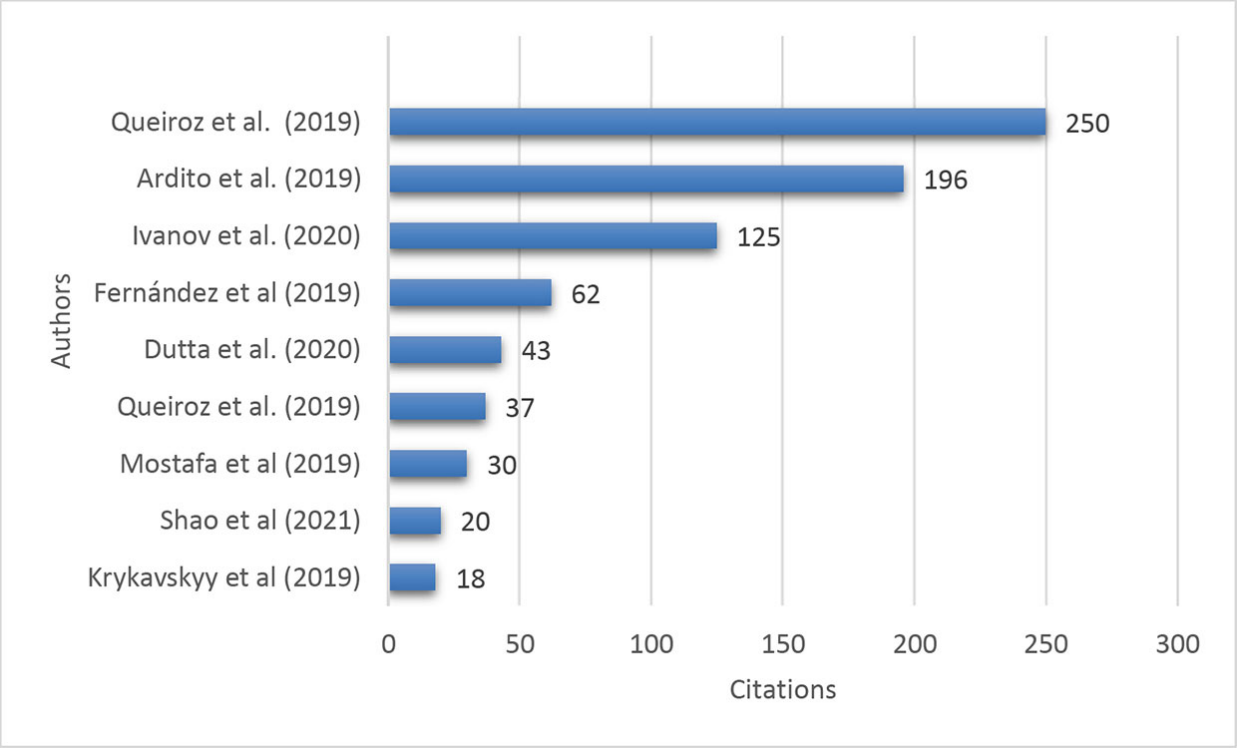
Top cited publications.

Figure 8 demonstrates the top-cited articles, and the publication by Queiroz ^41^ leads the list with 250 citations, followed by *Ardito, Petruzzelli, Panniello and Garavelli* ^7^ and Ivanov & Dolgui ^42^ with the second and third highest number of citations, 195 and 196, respectively. The citation trend shows that the research interest in Industry 4.0 technologies and supply chain management is growing as the other researchers cite these documents.

***High-contributing authors***

**Figure 9.**
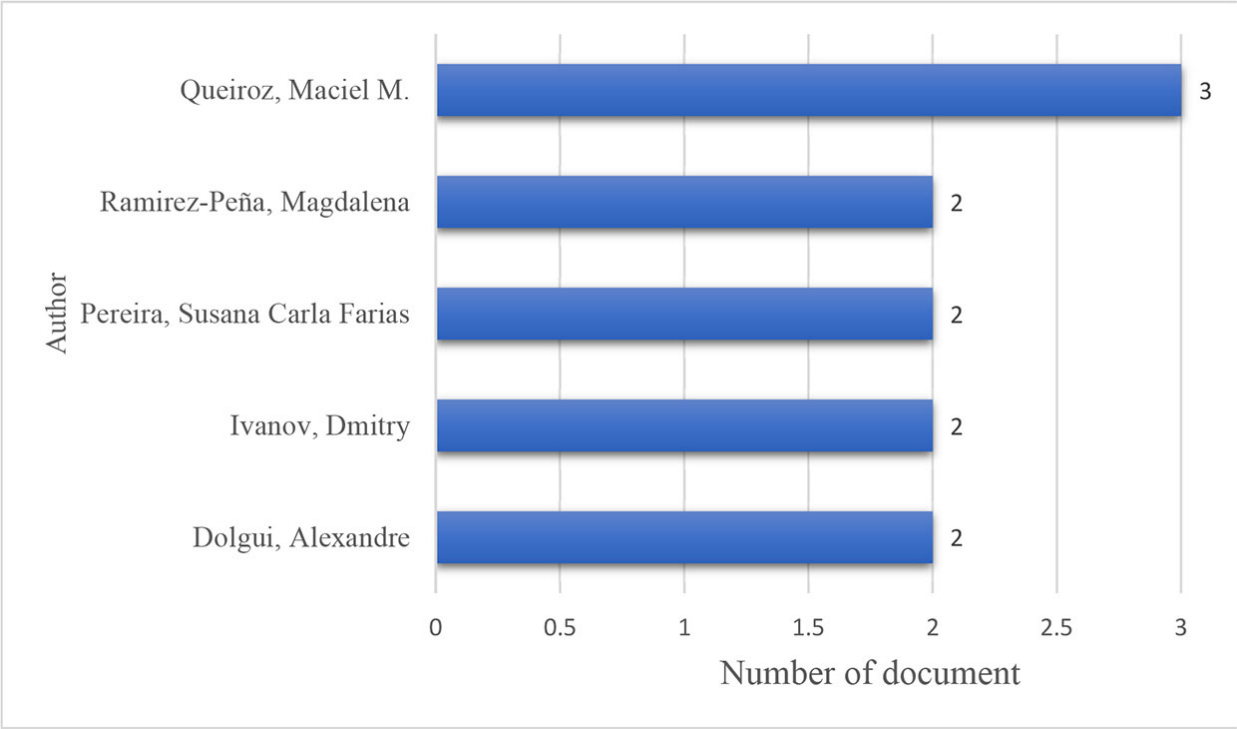
High contributing authors.

The list of high-contributing authors is shown in Figure 9. Queiroz tops the list with three publications, followed by Ramirez-Peña; Pereira; Dolgui, and Ivanov, with two publications each.

***Country-wise publications***

**Figure 10.**
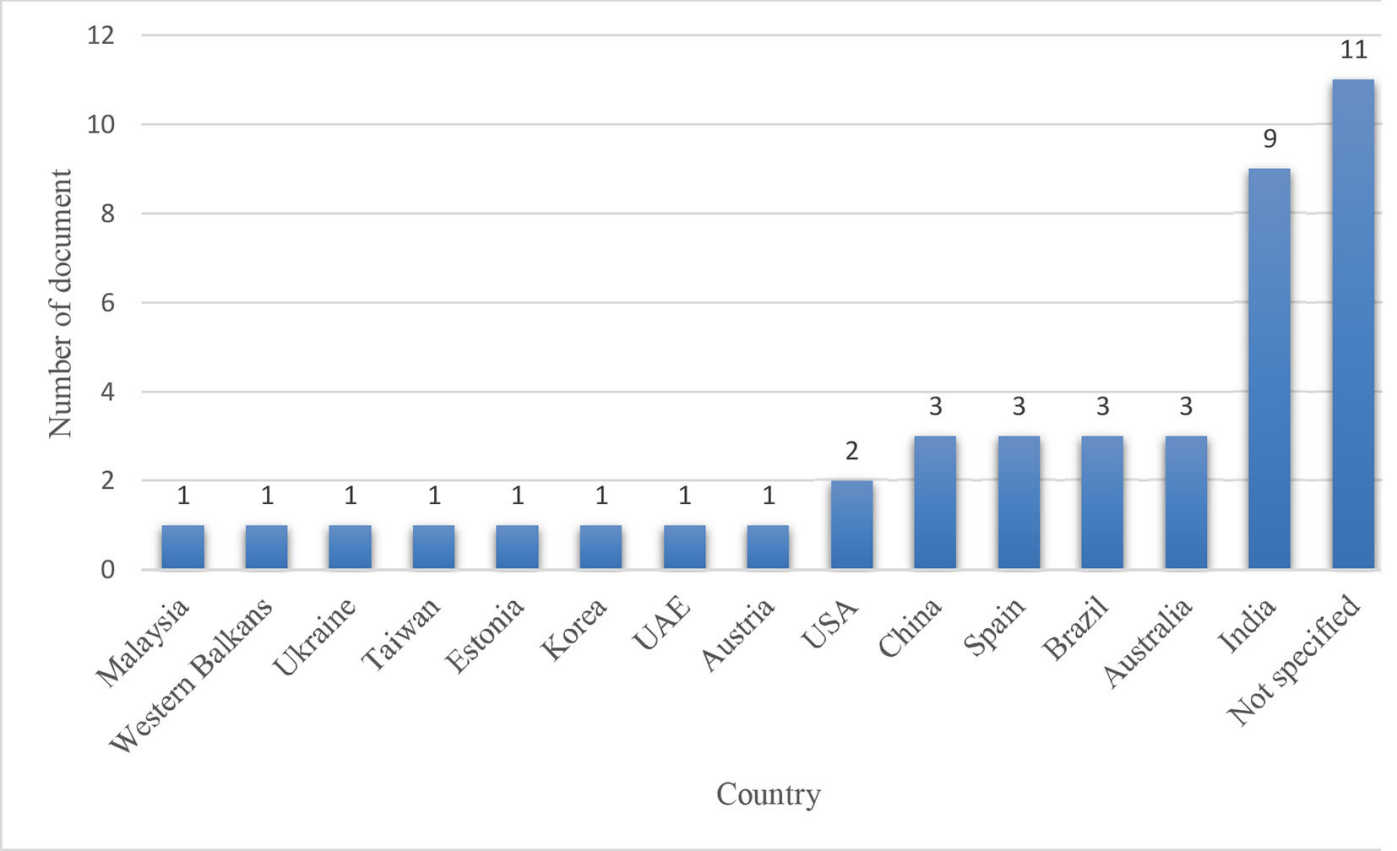
Country-wise distribution.

The authors’ affiliations with various countries were extracted. Figure 10 shows that India tops the list with ten out of the selected 42 articles, followed by Australia, Brazil, Spain, and China with three papers each. 2 documents were found published from the USA. Among the 42 reviewed publications, 15 did not explicitly mention the country, as many authors conducted systematic/general literature reviews or conceptual studies.

***Sector-wise distribution***

**Figure 11.**
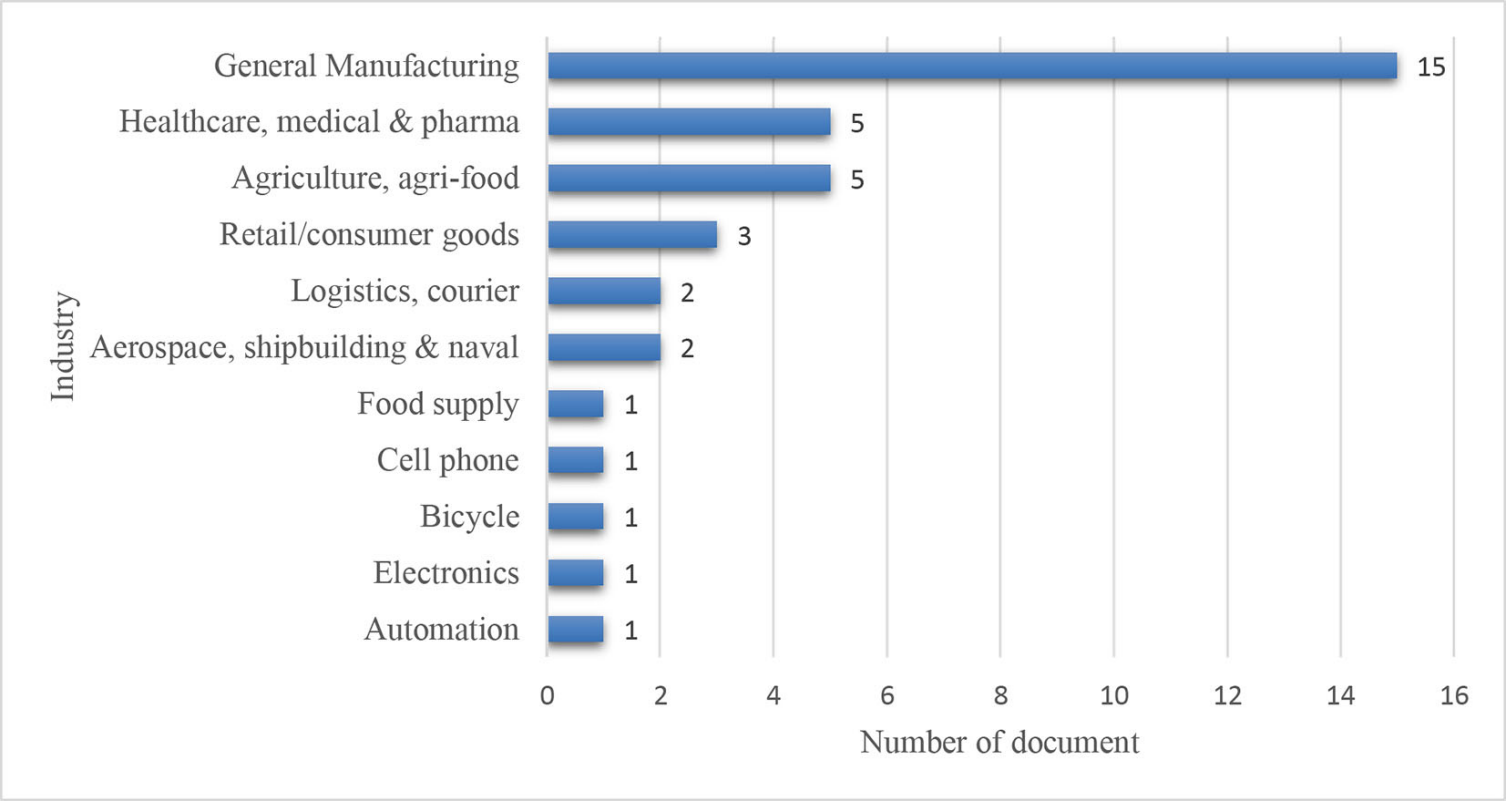
Sector-wise distribution.

Figure 11 illustrates the sector-wise distribution, and the manufacturing supply chains account for 15 articles out of 42. Healthcare, medical & pharmaceuticals, and agriculture & agri-food supply chains are reflected in each of the five articles. Three papers focused on retail/consumer goods supply chain. Each of the two articles emphasizes logistics/courier and aerospace, shipbuilding & naval supply chains.

***Identifying the emerging technologies for supply chain recovery***

**Figure 12.**
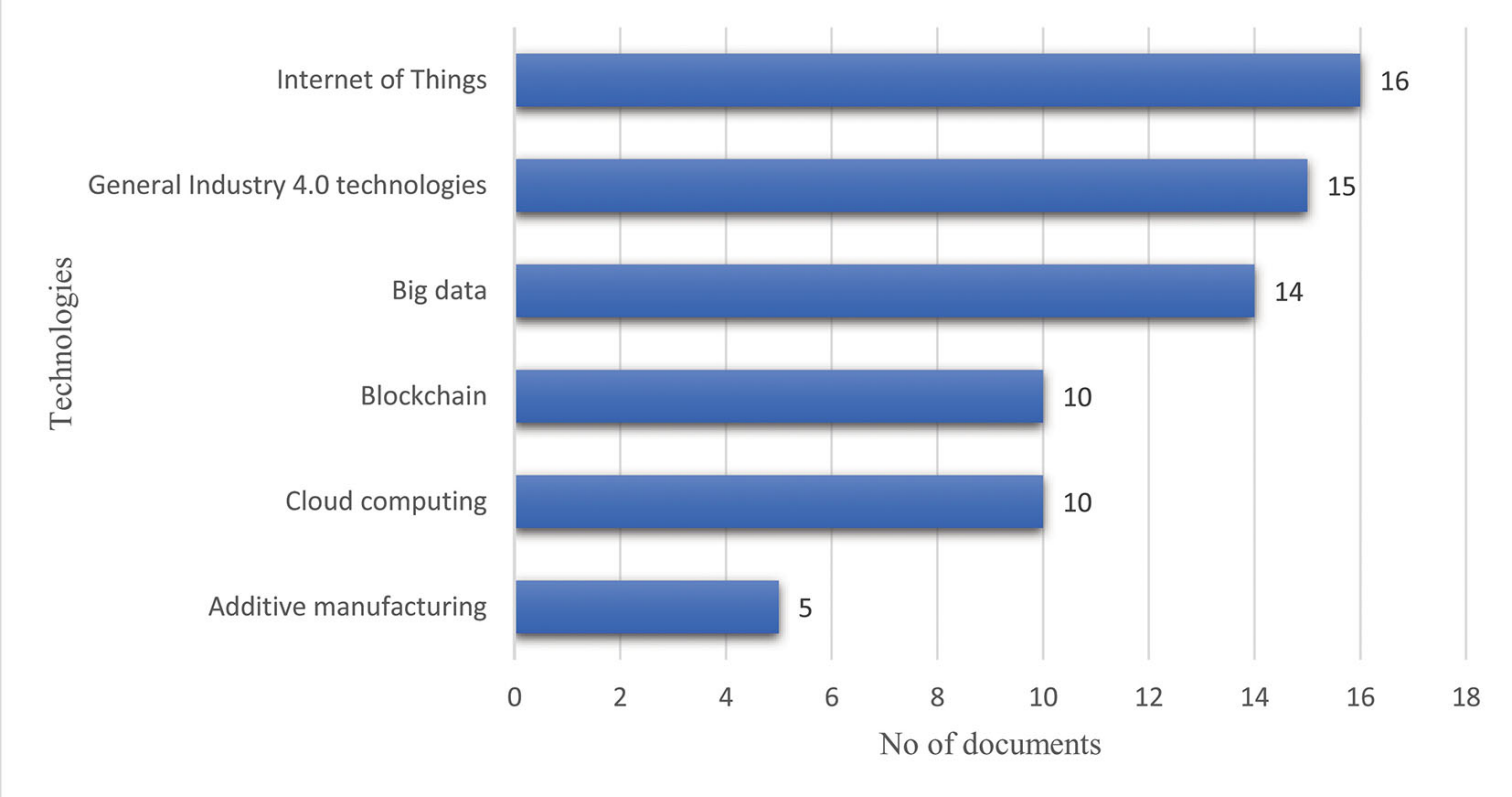
Frequency of the emerging technologies identified in the reviewed literature.

The selected articles were evaluated to recognize the technologies suggested by the authors for managing the supply chain disruptions caused by the COVID-19 pandemic. The literature review highlighted that emerging technologies would streamline supply chain resilience, resulting in increased robustness during an emergency or an unexpected and dynamic catastrophe. These technologies include the Internet of Things, big data, cloud computing, additive manufacturing, and blockchain. The results are summarized in Table 2 and Figure 12.

**Table 2.** The emerging technologies identified in the reviewed literature.

Technologies	References
General Industry 4.0 technologies	Frederico, ^1^ Acioli, ^6^ Shao, ^14^ Andiyappillai, ^30^ Brandtner, ^17^ Hopkins, ^43^ Ramirez-Peña, Abad Fraga, ^44^ Ramirez-Peña, Mayuet, ^45^ Wang, ^46^ Dolgui, Ivanov, Sethi, ^47^ Krykavskyy, ^48^ Queiroz, Machado, ^49^ Gu, ^19^ Li, ^50^ Quayson ^51^
Internet of Things	Mubarik, ^13^ Benitez, ^28^ Amer, ^37^ P.K. Dutta, ^52^ Galanakis, ^18^ Raji, ^27^ Yadav, ^15^ Chanchaichujit, ^53^ Haji, ^20^ Hossain, ^54^ Končar, ^55^ Kumar, ^56^ Ardito, ^7^ Chauhan, ^25^ Fernández-Caramés, ^23^ Mostafa ^31^
Big data	Mubarik, ^13^ Gupta, ^36^ Benitez, ^28^ Amer, ^37^ Brandtner, ^17^ Lv, ^54^ Raji, ^27^ Hossain, ^54^ Ivanov, ^41^ Ardito, ^7^ Chauhan, ^25^ Fernández-Caramés, ^23^ Mostafa, ^31^ Queiroz & Pereira ^57^
Cloud computing	Raji, ^27^ Chanchaichujit, ^53^ Haji, ^20^ Hossain, ^54^ Ivanov, ^41^ Kumar, ^56^ Ardito, ^7^ Chauhan, ^25^ Fernández-Caramés, ^23^ Mostafa ^31^
Additive manufacturing	Benitez, ^28^ Raji, ^27^ Haji, ^20^ Ardito, ^7^ Verboeket ^28^
Blockchain	Ahmad, ^58^ Galanakis, ^18^ Yadav, ^15^ Abbas, ^59^ P. Dutta, ^22^ Haji, ^20^ Ivanov, ^42^ Kumar, ^56^ Fernández-Caramés, ^23^ Queiroz & Wamba ^41^

The frequency of the emerging technologies identified in the reviewed literature is demonstrated in Figure 12. The result shows that the technologies were studied individually, collectively, or even as a bundle of general Industry 4.0 technologies. Notably, the Internet of Things (IoT) received a higher concentration to manage the supply chain disruptions, and it was explored in16 publications out of the 42. However, Industry 4.0 technologies were investigated as a bundle in 15 articles where the authors integrated all the emerging technologies and showed their applicability in managing supply chain disruptions. The potential of big data and its tools, techniques, and applications was demonstrated in 14 articles, followed by blockchain and cloud computing with ten articles each. The authors paid little attention to additive manufacturing. Only five publications exhibited the prospects and applications of this technology.

***Advantages of employing emerging technologies in supply chains during COVID-19***

COVID-19 emphasizes the whole production process and the structural advantages of integrating Industry 4.0 technologies, supply chains, and the COVID-19 pandemic. In this section, these advantages are identified, including supply chain monitoring, integration, and responsiveness, obtaining real-time data, warehouse management, and waste reduction. Table 3 illustrates the significant role and advantages of employing emerging technologies in supply chains, particularly in any disrupted situation. On the other hand, Table 4 summari **z**es the details of the literature review, including the focused technologies of Industry 4.0, research area, and approach. The table also demonstrates the findings of each of the studies.

**Table 3.** Advantages of employing emerging technologies in supply chains.

		Monitoring				Integration		Responsiveness		Real-time data		Warehouse management		Waste reduction	
SL	Author	Demand management	Operations planning	Product management	Risk management	Information sharing	Supplier and customer integration	Quick response	On-time delivery	Operational data	Logistics data	Warehouse design	Inventory management	Production line reviewing	Communications channel mapping
1	Acioli ^6^						x		x			x		x	
2	Frederico ^1^						x								
3	Mubarik ^13^				x		x		x				x		x
4	Gupta ^36^				x				x						
5	Benitez ^28^		x	x	x	x	x			x					
6	Shao ^14^		x	x	x	x	x			x					
7	Aamer ^37^			x	x									x	
8	Ahmad ^58^		x			x		x		x			x		
9	Andiyappillai ^30^	x	x			x		x			x		x		
10	Brandtner ^17^	x		x				x							
11	Choudhury ^26^	x	x	x	x	x		x	x	x	x	x	x		
12	Dutta ^52^														
13	Galanakis ^18^				x			x							
14	Gu ^19^	x	x			x	x	x	x				x		
15	Hopkins ^43^	x		x	x				x	x			x		
16	Lv ^60^	x				x		x	x				x		
17	Raji ^27^	x	x	x	x	x	x	x	x	x		x	x	x	
18	Yadav ^15^	x	x			x	x	x		x	x	x			
19	Abbas ^59^		x			x	x		x	x					
20	Arora ^61^	x											x		
21	Chanch ^53^	x		x		x	x			x		x	x		
22	P. Dutta ^22^	x	x	x	x	x				x	x	x		x	
23	Haji ^20^	x		x				x	x	x		x	x		
24	Hossain ^54^	x	x			x			x		x		x		
25	Ivanov ^42^	x	x		x	x		x	x	x	x		x		
26	Končar ^55^	x	x	x											
27	Kumar ^56^		x										x		
28	Li ^50^	x	x	x	x	x	x	x	x	x			x		
29	Quayson ^51^								x						
30	Ramirez-Peña ^45^		x	x	x						x		x		x
31	Ramirez-Peña ^44^		x												x
32	Wang ^46^				x	x		x	x		x		x		
33	Dolgui ^47^	x	x										x		
34	Ardito ^7^	x	x	x		x	x			x			x		
35	Chauhan ^25^		x	x											
36	Fernández-Caramés ^23^					x		x		x		x	x		
37	Krykavskyy ^48^		x												
38	Mostafa ^31^	x		x		x			x	x		x	x		
39	Queiroz ^57^														
40	Queiroz ^49^		x	x			x			x		x			
41	Queiroz ^41^		x		x	x									
42	Verboeket ^29^	x		x	x		x		x				x		x

**Table 4.** Summary of reviewed literature.

SL	Author	Technology	Research area	Research approach	Findings
1	Frederico ^1^	General Industry 4.0 technologies	Manufacturing	General review	The authors reviewed the prospects of Industry 4.0 technologies in collecting, utilizing, and generating data to establish a self-executed and controlled supply chain process during disruptions.
2	Acioli ^6^	General Industry 4.0 technologies	Manufacturing	Systematic literature review	The study analyses the potential of using Industry 4.0 technologies for sustainable supply chain performance during COVID-19.
3	Mubarik ^13^	IoT, Big data	Electronics	Empirical (survey)	During COVID-19, the study evaluated solutions to increase supply chain resilience and visibility by integrating consumers, suppliers, and related parties using Industry 4.0 technologies.
4	Gupta ^36^	Big data	Not specified	Conceptual	The authors demonstrated the potential of big data analytics in demand forecasting, sourcing, and development, maximizing resources and production output, on-time delivery, reducing costs, and increasing the supply chain transparency during COVID-19.
5	Benitez ^28^	IoT, Big data, Additive manufacturing	Automation	Empirical (survey)	The study revealed that employing Industry 4.0 technologies in the supply chain would increase the integration between customers and R&D canters, enhance customer loyalty, long-term technology innovation, and reduce costs.
6	Shao ^14^	General Industry 4.0 technologies	Packaging industry	Case study	The study proposed a multi-level implementation framework highlighting Industry 4.0 technologies implementation at multiple levels of the supply chain during disruption.
7	Amer ^37^	IoT, Big data	Food supply	Systematic literature review	The study revealed the prospects of IoT in the food supply chain during COVID-19 pandemic, uncovering the technical, financial, social, operational, educational, and regulatory challenges.
8	Ahmad ^58^	Blockchain	Healthcare and medical equipment	Prototype	The study proposed a decentralized blockchain-based solution to automate the supply chain enabling secure, transparent, traceable, and trustworthy data sharing between the stakeholders involved in waste management during COVID-19.
9	Andiyappillai ^30^	General Industry 4.0 technologies	Logistics	Interview	Focusing on the logistics and supply chains, the study illustrated the potential of Industry 4.0 technologies in improving inventory management, customer loyalty, shortening product cycles, and performing tasks rapidly during disruptions.
10	Brandtner ^17^	Big data	Retail industry	Data mining	Employing a data mining approach, the study revealed the prospects of big data in demand and store planning and control the supply chain in retail, increasing consumer satisfaction during COVID-19.
11	Brandtner ^26^	General Industry 4.0 technologies	Not specified	General review	The authors showed that the digital supply chain might improve sales and operations planning strategies, procurement techniques, intelligent manufacturing processes, and warehouse management in COVID-19.
12	P.K. Dutta ^52^	IoT	Agriculture	Prototype	Highlighting COVID-19, the study demonstrated the potential of IoT-based wireless sensors in agriculture, suitable for monitoring and monitoring seed, harvest, and similar packaging operations.
13	Galanakis ^18^	IoT, Blockchain	Agri-food & beverage	General review	Focusing on the food supply chain in COVID-19, the study examined the prospects of Industry 4.0 technologies, particularly in food safety, bioactive compound safety, and sustainability.
14	Gu ^19^	General Industry 4.0 technologies	Manufacturing	Empirical (survey)	The study demonstrated how companies implement Industry 4.0 technologies with supply chain partners to achieve supplier and customer resilience and empirically investigated the performance implications of these two dimensions on supply chain resilience.
15	Hopkins ^43^	General Industry 4.0 technologies	Manufacturing	Empirical (survey)	The authors documented that most Industry 4.0 technologies adoption is still in the early stages of supply chains.
16	Lv ^60^	Big data	Cell phone	General review	The study proposed a six-sigma method to optimize supply chain management and master customer demand through big data analysis and market research to reduce production costs and improve customer satisfaction.
17	Raji ^27^	IoT, Big data, Cloud, Additive manufacturing	Manufacturing	Empirical (survey)	Proposing a framework, the study demonstrated the linkage between Industry 4.0 technologies with lean and agile supply chain practices geared towards achieving the key performance indicators in the supply chain during disruptions.
18	Yadav ^15^	IoT, Blockchain	Agri-Food	Review/survey	The study proposed an IoT-based efficient and supportive coordination system to improve the operational mechanism of the food supply chain management in agriculture during natural outbreaks.
19	Abbas ^59^	Blockchain	Medical & pharmaceutical	Theoretical simulation	The authors proposed a blockchain and machine learning-based drug supply chain management and recommendation system to monitor and track the delivery process and recommend the best drugs for the customers.
20	Arora ^61^	Additive manufacturing	Medical & pharmaceutical	General review	The findings of the study showed that Industry 4.0 technologies enabled establishing the digital supply chain and shortened lead time to help fight COVID-19.
21	Chanchaichujit ^53^	IoT, Cloud	Not specified	Systematic literature review	The study identified six key drivers for implementing IoT and cloud technologies in the supply chain, focusing on how traceability helps meet customer demand leading to supply chain profitability.
22	P. Dutta ^22^	Blockchain	Manufacturing	Systematic literature review	The study summarized the key benefits of blockchain in supply chains, different uses of blockchain in various supply chain functions and operations, and their impact on society.
23	Haji ^20^	IoT, Cloud, Additive manufacturing, blockchain	Agri-food	Systematic literature review	The authors identified the different technological implementations in the food supply chain and pointed out the critical factors behind using technologies to improve the efficiency of the perishable food supply chain.
24	Hossain ^54^	IoT, Big data, Cloud	Healthcare	Theoretical simulation (new)	The study results showed that Industry 4.0 technologies should be employed to improve logistics management to establish a resilient healthcare supply chain.
25	Ivanov ^42^	Big data, Cloud, Blockchain	Manufacturing	Theoretical simulation	The study combined model-based and data-driven approaches to improve predictive and reactive decisions to take advantage of supply chain visualization and historical and real-time data analysis to ensure end-to-end transparency.
26	Končar ^55^	IoT	Consumer goods	Empirical (survey)	The study defines the setbacks in digitizing business processes through IoT and sustainability of the fast-moving consumer goods supply chain based on the six phases of product monitoring across the entire supply chain.
27	Kumar ^56^	IoT, Cloud, Blockchain	Health-care	Systematic literature review	The study highlighted the health sector’s challenges during COVID-19 and suggested potential solutions for supply chain management emphasizing Industry 4.0 technologies.
28	Li ^50^	General Industry 4.0 technologies	Bicycle	Empirical (survey)	The authors empirically revealed that Industry 4.0 technologies had significant positive effects on supply chain capabilities influencing operational and financial performance for the supply chain partners.
29	Quayson ^51^	General Industry 4.0 technologies	Agriculture	General review	The study suggested employing Industry 4.0 technologies in building resilient and sustainable supply chains, especially for smallholder farmers, to avoid significant disruptions caused by COVID-19.
30	Ramirez-Peña, Mayuet ^45^	General Industry 4.0 technologies	Aerospace, naval, and automotive	Systematic literature review	The study showed that the aerospace, marine, and automotive industries were showing a keen interest in Industry 4.0 technologies, which are of paramount importance for the sustainability of the supply chain.
31	Ramirez-Peña, Abad Fraga ^44^	General Industry 4.0 technologies	Shipbuilding	Systematic literature review	The authors emphasized employing Industry 4.0 technologies to achieve the economic, ecological, and social factors of supply chain management in establishing a long-term and sustainable supply chain.
32	Wang ^46^	General Industry 4.0 technologies	Courier & delivery services	Empirical (survey)	The study empirically proved that Industry 4.0 technologies would enable logistical innovation capacity to reduce different types of supply chain risks, including corporate, customer, and environmental challenges.
33	Dolgui ^47^	General Industry 4.0 technologies	Manufacturing	Theoretical simulation	The study offered an effective operations model for job and flow shop planning in production, supply chain, and Industry 4.0 technologies.
34	Ardito ^7^	IoT, Big data, Cloud, Additive manufacturing	Manufacturing	Patent analysis	The study highlighted Industry 4.0 technologies integrating the supply chain and marketing interface from information processing to collect and share marketing and operational data between marketing functions and the supply chain.
35	Chauhan ^25^	IoT, Big data, Cloud	Manufacturing	Systematic literature review	The authors demonstrated Industry 4.0 technologies, concerns, enablers, and impacts on various supply chain approaches and performance.
36	Fernández-Caramés ^23^	IoT, Big data, Cloud, Blockchain	Manufacturing	General review	The study presented an “unmanned aerial vehicle-based system” for automating inventory tasks capable of collecting inventory data faster than human operators and locating items in the warehouse using their tags signal.
37	Krykavskyy ^48^	General Industry 4.0 technologies	Retail Industry	Empirical (survey)	The authors demonstrated the effects of Industry 4.0 technologies in a cross-section of strategic and operational changes in the supply chain. They clarified the technological readiness and capability among the organizations.
38	Mostafa ^31^	IoT, Big data, Cloud	Manufacturing	Conceptual	The study proposed an IoT-based framework for warehouse management, providing a real-time overview of stocks, increasing speed and efficiency, prevent inventory bottlenecks and counterfeiting.
39	Queiroz & Pereira ^57^	Big data	Manufacturing	Empirical (survey)	The authors underlined the importance of big data in supply chain management and suggested developing IT infrastructure influencing the intention to adopt big data.
40	Queiroz, Machado ^49^	General Industry 4.0 technologies	Manufacturing	Conceptual	The study proposed a framework encompassing six major Industry 4.0 technologies and seven fundamental skills for the digital supply chain management.
41	Queiroz & Wamba ^41^	Blockchain	Manufacturing	Empirical (survey)	The study shed light on blockchain implementation behaviour in supply chain management and showed that blockchain implementation by logistics and supply chain management experts is still in its infancy.
42	Verboeket ^29^	General Industry 4.0 technologies	Manufacturing	Systematic literature review	The study documented that the properties of additive manufacturing, such as design flexibility and complexity and the absence of object-specific tools, would optimize supply chain design for manufacturing purposes.

## Discussion

The categorical analysis, including the cluster and sub-cluster levels, is presented in the following section. The section concludes by highlighting the research gaps addressed in the reviewed literature.

### Phase 5. Categorical analysis

**Figure 13.**
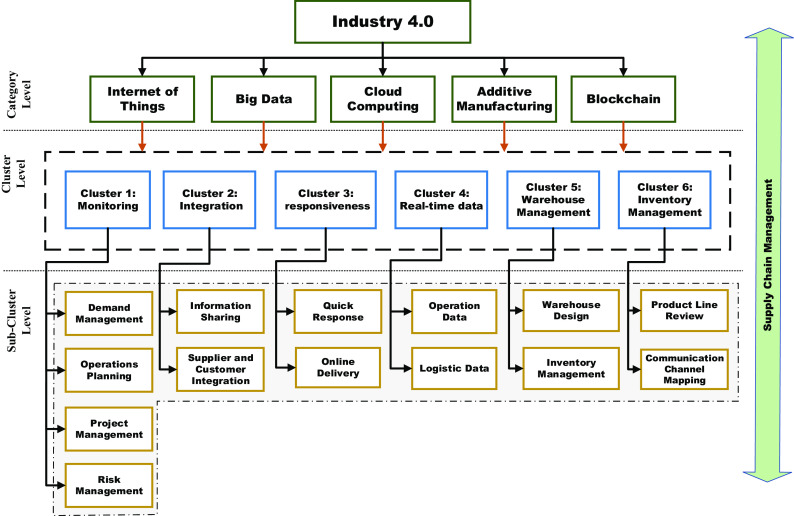
Identification of category, clusters, and sub-clusters.

COVID-19 underlines the entire manufacturing system and the structural aspects linking Industry 4.0 technologies, supply chains, and the pandemic itself. The current review investigated the main topics concerning Industry 4.0 technologies and the prospects of implementing these technologies in supply chains to recover the disruptions caused by the COVID-19. The following section illustrates the findings of the categorical analysis and answers the two research questions. According to the reviewed literature Figure 13, six significant advantages (clusters) have been identified that could be achieved by deploying Industry 4.0 technologies in supply chains during COVID-19. The advantages include supply chain monitoring, integration, and responsiveness, obtaining real-time data, warehouse management, and waste management. In Figure 13, Industry 4.0 technologies are presented as the main categories, and other clusters and related sub-clusters are also illustrated. The classification followed a thorough interpretation of all identified articles and categorizing them. The identified clusters represent macro-categories, summarizing the top-level concepts discussed in the papers. Sub-clusters identify successive partitions of a cluster and are used to specify more detailed subjects of the scientific literature. The proposed categorization in clusters and sub-clusters and the relationship of a sub-cluster with a cluster are also illustrated.

***Cluster 1. supply chain monitoring***

The reviewed literature emphasise significant prospects and solutions for supply chain monitoring, and it was considered as the first cluster of categorical analysis. The authors classified this cluster into four sub-clusters, i.e., demand management, operations planning, product management, and risk management. The sub-clusters are discussed in the following sections.

**Sub-cluster 1: Demand management**

Customer demand management is the major challenge retailers face due to the COVID-19 outbreak. However, the literature showed that the emerging technologies have full potential to manage customer demand during a crisis. Choudhury ^26^ and Brandtner ^17^ showed that integrating Point of Sales (PoS) data in the form of Big Data offers a comprehensive view of customer-level understanding and company-level optimization for supply chain management both in normal and pandemic times. Cloud-based data collection and analysis for demand management were demonstrated by Raji. ^27^ Andiyappillai ^30^ proposed automated supply chain management to manage customer demand in real-time by capturing and evaluating essential data across the supply chain. Lv and Li ^60^ reviewed the six sigma management strategy to master customer demand through big data analysis. Highlighting the agri-food supply chain, Yadav ^15^ presented a coordination framework based on IoT. Haji ^20^ showed the potential of emerging technologies in the perishable food supply chain to meet the customer demand in any disruption during Tsunami or COVID-19. Arora ^61^ showed different additive manufacturing techniques in the medical and healthcare sectors. Verboeket and Krikke ^29^ presented additive manufacturing-based supply chain mechanisms. Ivanov ^42^ proposed a digital twin concept providing network states in real-time to analyze and meet the disrupted customer demand. Ardito ^7^ demonstrated that the planning of market and supply-focused initiatives simultaneously through Industry 4.0 technologies is essential to meet customer demand during any disruption.

**Sub-cluster 2. Operations planning**

Digital sales and operation planning strategies are essential for resilient supply chain management during COVID-19. ^26^ Thus, using Industry 4.0 technologies in the supply chain communication system enables supply chain capabilities. ^50^ Ahmad ^58^ advised employing emerging technologies in the supply chain, allowing operational transparency and efficient planning in healthcare and medical equipment manufacturing during COVID-19. Gu ^19^ studied that a solid external IT linkage with the supply chain partners enabled Haier to efficiently maintain the daily production and sales planning during the pandemic. To increase operational performance, Raji ^27^ reviewed the impact of Industry 4.0 technologies on lean and agile processes. Yadav ^15^ proposed an IoT-based coordinating framework to enhance sales and operational dynamism in the agri-food supply chain under natural outbreaks. Abbas ^59^ anticipated that blockchain-based drug supply chain management enabled customers to send update requests to the firm. Based on that, the firm could plan the sales and operational activities. Ivanov ^42^ proposed dynamic simulation models to analyze supply chain behavior and disruption performance based on real-time data for effective sales and operational planning. Ardito ^7^ discussed that the deployment of the emerging technologies in establishing a digital supply chain empowered Wal-Mart to enjoy operational and marketing efficiency. Chauhan ^25^ found that Industry 4.0 technologies provide rapid and cost-effective responsiveness by boosting supply chain flexibility, leading to enhanced overall operational planning.

**Sub-cluster 3. Product management**

Studies have shown that implementing Industry 4.0 technologies can improve supply chain efficiency, including product visibility, real-time tracking, and product quality guarantee. For product tracing, Benitez ^28^ mentioned digital devices, such as RFID, QR codes, whereas Yadav ^15^ and Choudhury ^26^ pointed to RFID tags, NFC chips, and GPS modules. Hopkins ^43^ showed that supply chain digitization empowers identifying counterfeit products and customization, shortening product development cycles, prototyping new products, and thus allowing product development monitoring remotely even in any crisis. Shao ^14^ also supported the same view. The study conducted by P. Dutta ^22^ showed that blockchain enables product management by certifying, tracking, and verifying products across the supply chain. Končar ^55^ showed the potential of IoT for linking product management and service system data that empowered the retail supply chain to monitor the whole supply system. This IoT-based system would offer complete transparency, efficiency, and product monitoring for better safety and security in any disaster. Ramirez-Peña, Mayuet ^45^ explored that aerospace companies developed smart supply chains and product life management systems by supervising the entire product life cycle, empowering them to operate their business in any disruption.

**Sub-cluster 4. Risk management**

Ardito ^7^ identified cyber security as a great threat to integrated supply chains and marketing functions. The authors recommended investing adequate capital in strengthening cyber security to protect all data flows in the supply chain. Mubarik ^13^ emphasized establishing the supply chain visibility to collect and evaluate supply chain data through employing emerging technologies. The authors also empirically tested the significant impact of supply chain visibility in risk management in any disruption, even in COVID-19. Benitez ^28^ advised collaboration with research & development centers in supply chain risk management to guide technological transformation by affording high-risk ventures. Aamer ^37^ identified data security and trust as significant concerns among the supply chain partners and data owners. To overcome this challenge, the authors suggested building a solid defense by deploying emerging technologies across the supply chain reinforcing cyber security, access control, data authentication, and customer privacy protection. To avoid inventory shortages or to carry more due to unreliable suppliers, Choudhury ^26^ studied digital supply chains for warehouse tracking with transit process tracing, improving security, and reducing supply-related risk. As a result of the COVID-19 pandemic, Galanakis ^18^ investigated the prospects of Industry 4.0 technologies to reduce supply chain management risks in food safety and security. Hopkins ^43^ and Li ^50^ advised employing Industry 4.0 technologies to analyze real-time fraud and risk management in supply chains. Raji ^27^ proposed an integrated lean and agile strategy termed “form postponement” for risk mitigation by delaying the ultimate form of a product until a consumer places an order. According to P. Dutta, ^22^ using blockchain on a double chain formula improves the visibility and security of transactions and the privacy and security of organizational data in supply chain management. Wang ^46^ empirically examined that organizations could reduce supply chain risks by building innovation capability by employing Industry 4.0 technologies in supply chain and logistics.

***Cluster 2: Supply chain integration***

The literature highlighted that inter-organizational information sharing and integration between supplier and customer are essential for supply chain integration. Supply chain integration was selected as the second cluster of the analysis and divided into two sub-clusters. The following discussion portrays the role and impact of Industry 4.0 technologies in enabling effective information sharing and integration across the supply chain during any disruption.

**Sub-cluster 1. Information sharing**

The implementation of Industry 4.0 technologies tends to improve the performance of the entire supply chain, mainly through information sharing, which enables greater agility, efficiency, and cost reduction. Information sharing in the supply chain would further aid in developing visibility and robust design, production performance, adaptability, and eliminating wastages. ^26^ Chanchaichujit ^53^ mentioned information sharing strengthens trust and collaboration among the supply chain members and enables capturing vital information to improve consumer satisfaction during any disruption. Shao ^14^ suggested investing in IoT devices to enhance information sharing. Dutta ^22^ advocated using blockchain and big data in agriculture and food supply chains. Ahmad ^58^ proposed a decentralized blockchain-based model to automate supply chain processes for COVID-19 medical equipment distribution. The model would enable information exchange among stakeholders, assuring a fully secure, seamless, visible, and integrated supply chain management system. Gu ^19^ emphasized sharing standardized information formats in supply chains, allowing the supply chain partners to respond swiftly to any uncertainty. Hossain ^54^ proposed an information-sharing system to match demand and supply for medical products while increasing inventory visibility. Using a supply-chain management system, Li ^50^ offered a model for enhancing supply-chain capabilities in data sharing. According to Ardito, ^7^ cloud computing would assist in preparing various structured information and sharing, decreasing the need for multifaceted and customized IT systems within the organization and across the supply chain. Fernández-Caramés ^23^ proposed “Unmanned Aerial Vehicles,” capable of storing, processing, and exchanging information with suppliers and factory-based devices.

**Sub-cluster 2. Supplier and customer integration**

The supply chain would not be effective without integrating supplier and customer. ^14^^,^^27^ In this regard, the emerging technologies enable integration between supplier and customer, establishing a trustworthy relationship and resilient supply chain. ^6^^,^^28^ Frederico ^1^ proposed an Industry 4.0 technology-integrated supply chain named “Supply Chain 4.0”, able to share real-time information among the suppliers, customers, and stakeholders, enhancing the supply chain resilience, creating more robustness amid an emergency. Mubarik ^13^ introduced supply chain mapping in visualizing suppliers’ and consumers’ business activities by tracking upward and downward supply chain flows, including information, resources, funds, and across the firm. Technology-based integration between suppliers and customers allows firms to monitor the changes in demand. In contrast, customers can track supply and logistics data over time, enabling both parties to take rapid action in abnormal signals indicating possible disruptions. ^19^ Abbas ^59^ proposed a blockchain-based “drug supply chain management and recommendation system” integrating the suppliers, customers, and other stakeholders to perform any activity across the supply chain. Li ^50^ stated that Apple, Ford, and Walmart had established an effective supplier and customer integration system to make the supply chain resilient during any emergency. Ardito ^7^ referred to planning, forecasting, and replenishment practice to estimate demand and quantities for existing items by integrating suppliers and customers across the supply chain. The digital supply chain capabilities framework offered by Queiroz ^49^ investigated the influence of supplier and customer integration on the whole supply chain.

***Cluster 3. Responsiveness***

Supply chain responsiveness is vital for customer satisfaction. The literature reported several challenges related to supply chain responsiveness caused by the COVID-19. Supply chain responsiveness is identified as the third cluster of the analysis, including two sub-clusters. The following section illustrates how Industry 4.0 technologies enable supply chains to respond to customers quickly and deliver products on time during any disruption.

**Sub-cluster 1. Quick response**

Brandtner ^17^ recommended a digital supply chain, the most effective way to satisfy customer demand quickly. Choudhury ^26^ found the quick response to customer demand as one of the significant enablers of improving customer satisfaction. Ahmad ^58^ proposed a tech-based forward supply chain to respond quickly to customer demand during COVID-19. Galanakis ^18^ showed that using data analytics and smart technologies, including remote or virtual inspections, will shorten response time to foodborne outbreaks. According to Gu, ^19^ a well-balanced and complimentary usage of IT patterns in supply chains empowers to create standardized and simplified operations to respond rapidly during any disruption. Raji ^27^ studied lean and agile supply chain approaches and proposed enabling real-time data collection, integration, and sharing using cloud technology to respond swiftly. Yadav ^15^ highlighted IoT devices, third-party logistics companies, quick transactional responses between and within companies, rapid sharing of real-time information would improve the flexibility of the agri-food supply chain during disruptions. Haji, ^20^ offered the digital technology-based “unnamed aerial and autonomous vehicles,” enhancing speed and responsiveness to meet customers’ demands in the perishable food supply chain.

**Sub-cluster 2. On-time delivery**

In the current volatile market, customer satisfaction highly depends on the timely delivery of the ordered products. Literature shows that digital supply chains would empower in improving on-time delivery of the products. Mubarik ^13^ mentioned supply chain mapping for timely delivery. Choudhury ^26^ demonstrated the potential of additive manufacturing to create products directly at distribution hubs to speed up and strengthen the supply chain. Gu ^19^ offered a concept in which upstream and downstream partners used IT to share information efficiently and deliver properly in the supply chain during any disruption. Hopkins ^43^ stated IoT-based drones; Raji ^27^ and Haji, ^20^ pointed drones and aerial vehicles, and unmanned aerial vehicles, respectively, for the quickest delivery at any emergency. Abbas ^59^ introduced a blockchain-based Hyperledger fabric for secure drug supply chain management to track the drug delivery process to resolve counterfeiting issues and make the delivery faster. Li ^50^ reviewed digital supply chains with the integrated production plan, smart forecasting of activities, and production procedures leading to the decreased number of wrong deliveries and enhanced on-time delivery. Quayson ^51^ mentioned that smallholder farmers’ investments might be protected from significant disruptions by employing Industry 4.0 technologies in the supply chains and delivering the products to the customer on time. Mostafa ^31^ proposed a framework for warehouse performance improvement, inventory shortage prevention, and timely delivery of the products.

***Cluster 4. Real-time data***

Real-time data acquisition throughout the supply chains would enable organizations to recognize new ways to improve their practices during any disruptions. Real-time data collection is considered the fourth cluster of the categorical analysis and includes two sub-clusters. The following section demonstrates the role of Industry 4.0 technologies in empowering organizations to collect real-time data, assisting them in retaining sustainable during natural outbreaks.

**Sub-cluster 1. Operational data**

Industry 4.0 technologies enable firms to collect real-time data for business operations, allowing for higher traceability, monitoring, better prediction, strategic planning, enhanced product customization, reduction in inadequacies, and enhanced automation of tedious activities. ^43^ Yadav ^15^ presented an IoT-based agri-food supply chain enabling rapid real-time data sharing, improving responsiveness and resilience, and strengthening the adaptability of supply chains during COVID-19. Chanchaichujit ^53^ investigated pre-packaged food supply chains and concluded that technology-based tracking platforms enable real-time data collection and improve information-sharing, product quality, and safety. Dutta ^22^ inspected that Industry 4.0 technology ensured minimal delays in real-time transactions, enhanced visibility of transactions from remote places, additional vigilance for data protection, transaction authentication, and fraud control during disruptions. Haji, ^20^ reviewed real-time tracking technologies allowing more accessible updates to the shipping process, tracking orders and analyzing demands, identifying, locating, and monitoring the items’ status in the supply chain. Li ^50^ acknowledged real-time data collection through the digital supply chain allowing the members to allocate their resources. Ardito ^7^ revealed that Industry 4.0 technologies enable real-time customer data acquisition, product-customer communications, product life-cycle, and material flow to make rapid interactions across the supply chain. Fernández-Caramés ^23^ proposed a blockchain-based system for collecting and processing real-time inventory data, offering improved cyber security and resilience and operations of decentralized applications.

**Sub-cluster 2. logistics data**

Andiyappillai ^30^ evaluated automation and its implications on logistics. The authors advised using Industry 4.0 technologies to consolidate logistics activities by real-time data sharing. Choudhury ^26^ highlighted that the Industry 4.0 technologies enable real-time tracking and tracing to identify current and previous locations of specific items improving logistics capabilities. Yadav ^15^^,^^62^ analyzed third-party service providers to monitor relevant operational processes during any disruption using real-time visibility and traceability of food processing and logistics data. P. Dutta ^22^ revised the prospects of Industry 4.0 technologies in logistics for real-time data collection to establish resilient supply chain management. Hossain ^54^ proposed real-time healthcare logistic services to build capacity in supply chain management.

***Cluster 5. Warehouse management***

The fifth cluster of the analysis is warehouse management, and it is divided into two sub-clusters. The following discussion shows how Industry 4.0 technologies strategically establish resilient supply chains by improving warehouse design and inventory management to meet customer demand, even in pandemics.

**Sub-cluster 1. Warehouse design**

Acioli ^6^ reported that Industry 4.0 technologies enable real-time data acquisition to select parts from the warehouse for supply chain management during COVID-19. Choudhury ^26^ suggested using the big data-enabled forklift truck as the forklift vendors could track various warehouse operations in real-time. The authors also proposed employing robots for efficient supply chain and warehouse management techniques. Drones/aerial vehicles were introduced by Raji ^27^ to support performing cycle count or inventory checks on the manufacturing floor or warehouse. Yadav ^15^ advised tech-based “Third-Party Logistics Service Providers” for tracing, delivery scheduling, and distribution in real-time. Chanchaichujit ^53^ showed that Industry 4.0 technologies in warehouse management could help prevent counterfeit items and shoplifting. Dutta ^22^ studied the potential of blockchain in inbound, outbound, and reverse logistics to improve visibility with security, strengthen green marketing through innovative and traceable packaging, track the location of the products, and verify all parties engaged in the recycling system. Fernández-Caramés ^23^ presented the design, installation, and assessment of a blockchain-based technique. It was able to collect inventory data significantly quicker than a human operator and track products in the warehouse by using the signals of the products’ tags. Mostafa ^31^ proposed an IoT-based warehouse management system providing real-time visibility of all the products and warehouse functions, enhancing proficiency and eliminating inventory shortages.

**Sub-cluster 2. Inventory management**

Industry 4.0 technologies enable organizations to track upstream supply chain processes, allowing better inventory management during disruptions. ^13^^,^^30^ Shao ^14^ examined IoT and cloud technology to reduce inventory levels and expenditures to improve operational efficiency. Ahmad ^58^ offered blockchain-based decentralized storage of interplanetary file systems to safely collect, preserve and exchange data linked to the supply chain and inventory management. Choudhury ^26^ reviewed how digital supply chain agility helps avoid risks of running out of inventory or carrying extra to substitute for inconsistent suppliers and sync inventory level and sales objectives. A study by Chanchaichujit ^53^ examined the advantages of smart inventory management, including physical inventory counts, reduced procurement and inventory shortage expenses, improved inventory precision, and reduced inventory inconsistency. These advantages contribute to lowering inventory expenses, including other holding and ordering expenses in the supply chains.

***Cluster. Waste reduction***

The sixth and final cluster identified through the literature review is waste reduction. The cluster is classified into two sub-clusters. The following section demonstrates further explanations on the role and influence of Industry 4.0 technologies in reducing and preventing wastages along the supply chains.

**Sub-cluster 1. Review production line**

Industry 4.0 enables manufacturing process automation that reduces the process stages, material waste, and financial costs, as claimed by Acioli. ^6^ Proposing the IoT network system, Aamer ^37^ suggested automating waste management processes such as weighing food waste. The authors also highlighted IoT as a promising technology to avert data inaccessibility and discrepancy to measure and monitor food damage and waste across the food supply chain. Raji ^27^ recommended adopting lean and agile supply chain approaches to eliminate waste and non-value-adding items. These techniques would enable quick decision-making via a rapid data exchange over the cloud platform, avoiding waste caused by inappropriate communication and data discrepancies between suppliers and manufacturers. Dutta ^22^ highlighted the use of blockchain to reduce waste by effectively tracing materials.

**Sub-cluster 2. Mapping the communications channel**

Mubarik ^13^ found that supply chain mapping would help reduce the amount and toxicity of all pollutants and wastes at the source across the supply chain. Supply chain mapping could also assist in extending machine life, minimizing industrial waste creation, enhancing the use of local resources, and increasing the use and value of existing assets. Ramirez-Peña, Mayuet ^45^ proposed blockchain-based closed-loop supply chain models to improve traceability and transparency, increase trust with stakeholders, and reduce waste by setting up a collaborative program.

***Research gaps addressed in the literature***

The authors summarized the research gaps addressed in the reviewed articles as follows:

▪ It has been noted that the majority of articles have discussed supply chain management issues such as efficiency, technologies, and managerial procedures without integrating their roles in natural outbreaks or disruptions. There have been few studies on theory-based supply chain management systems ^15^^,^^59^ that can withstand natural disasters. This lack of research has hampered improving the supply chain resilience in any natural disruption to manage customer demand with safety and security.
▪ More than half of the reviewed articles conceptually integrated Industry 4.0 technologies and supply chain management, considering the disruptions caused by COVID-19 or natural outbreaks. Many conceptual studies address the lack of analytical research and demonstrate the absence of technical knowledge in this field.
▪ A significant number of studies emphasized that data security is a great concern for the safety and security of supply chain members/stakeholders due to a lack of secure online interfaces, poor software defense, and inadequate authorization. So, future research can focus on this issue.
▪ Many organizations continue to establish their manufacturing and supply chains, emphasizing the economies of centralized facilities. However, the integration process of sustainability aspects into the supply chain management is not properly developed yet. Therefore, researchers can focus on integrating supply chain management and sustainability to contribute to sustainable development goals.
▪ A holistic approach for evaluating and documenting the digital supply chain performance during COVID-19 is missing. Few studies ^55^^,^^59^ were identified addressing the issue of performance measurement in the context of Industry 4.0 and supply chain management. Research in the future may focus on developing methods for monitoring the performance of digital supply chains.
▪ The tools, techniques, and technologies of Industry 4.0 are associated with high investments and complex reconfiguration. It is a major concern among most organizations planning to establish digital supply chains. Hence, researchers should focus on future comprehensive studies encompassing the framework to investigate the financial viability of implementing Industry 4.0 technologies in supply chains.

### Limitations and future studies

The authors note several limitations in the study. First, the findings are derived considering English language-publications only, and those written in other languages are excluded. Future research may provide additional insights by reviewing the literature written in other languages. Second, the authors focus on the literature only in the context of Industry 4.0. Thus, the holistic view of Industry 4.0 has not been evaluated in this study. Furthermore, the study reviewed the role of the five major technologies such as IoT, big data, cloud computing, additive manufacturing, and blockchain and discussed how these technologies could be employed to revive the supply chains during emergencies. Future studies may include other emerging technologies such as artificial intelligence, robotics, augmented reality, and simulation/digital twins to get a broader range of findings. In spite of these constraints, the current study adds to the identification of significant technologies and their roles in supply chain management in the area of Industry 4.0.

## Conclusion

Recent studies have emphasized the impact of individual technologies on the supply chain, such as IoT, big data, cloud computing, additive manufacturing, and blockchain, and how these technologies support companies in achieving competitive advantage. However, comparatively few studies have explored the influence of these technologies concurrently, particularly during an unexpected situation. The present study is based on these gaps and responds to the research questions using a systematic literature review. In answering the first research question, the study confirmed that most publications highlight IoT, big data, cloud computing, additive manufacturing, and blockchain that may assist in building resilient and robust supply chains, even in the COVID-19 era. Regarding the second research question, the categorical analysis of the study indicates that the roles and functions of these technologies would lead to establishing integrated, flexible, responsive, and efficient supply chains enabling remote monitoring, operation, and inspection during the pandemic. The study also reveals unexplored features of supply chains. Therefore, highlighting a discussion on implementing Industry 4.0 technologies in supply chain studies offers an interesting future research topic.

## Data availability

### Underlying data

All data underlying the results are available as part of the article, and no additional source data are required.

### Reporting guidelines

Figshare: PRISMA Checklist_The Implications of Industry 4.0 on Supply Chains Amid the Covid 19 Pandemic – A Systematic Literature Re.docx, https://doi.org/10.6084/m9.figshare.16602356.

Data are available under the terms of the Creative Commons Attribution 4.0 International license (CC-BY 4.0).
