# Supplementary material for: The implications of Industry 4.0 on supply chains amid the COVID-19 pandemic: a systematic review
Source: F1000Res. 2022 Jan 31;10:1008. Originally published 2021 Oct 5. [Version 2] doi: 10.12688/f1000research.73138.2 (PMC8961196; doi:10.12688/f1000research.73138.2)
Supplement: Peer review history

# Peer review: Reviewer response for version 2

**DOI:** 10.5256/f1000research.119838.r121549

**Published:** 2022-03-28

**Authors:** Lorenzo Ardito

**Affiliations:** 1 Dipartimento di Meccanica Matematica, Management Ringgold Standard Institution, Politecnico di Bari, Bari, Italy

The authors, in compliance with the word limit, have addressed the points I raised. The paper can be accepted.

Are the rationale for, and objectives of, the Systematic Review clearly stated?

Partly

Is the statistical analysis and its interpretation appropriate?

No

Are sufficient details of the methods and analysis provided to allow replication by others?

Partly

Are the conclusions drawn adequately supported by the results presented in the review?

No

Reviewer Expertise:

Innovation, digital transformation

I confirm that I have read this submission and believe that I have an appropriate level of expertise to confirm that it is of an acceptable scientific standard.

# Peer review: Reviewer response for version 2

**DOI:** 10.5256/f1000research.119838.r121548

**Published:** 2022-02-08

**Authors:** Guilherme Brittes Benitez

**Affiliations:** 1 Department of Industrial Engineering, Federal University of Rio Grande do Sul, Porto Alegre, Brazil

I believe this new version is now suitable for indexing at F1000Reseach.

Are the rationale for, and objectives of, the Systematic Review clearly stated?

Yes

Is the statistical analysis and its interpretation appropriate?

Not applicable

Are sufficient details of the methods and analysis provided to allow replication by others?

Yes

Are the conclusions drawn adequately supported by the results presented in the review?

Partly

Reviewer Expertise:

Industry 4.0 and supply chain.

I confirm that I have read this submission and believe that I have an appropriate level of expertise to confirm that it is of an acceptable scientific standard.

# Peer review: Reviewer response for version 1

**DOI:** 10.5256/f1000research.76766.r96186

**Published:** 2021-11-15

**Authors:** Chetna Chauhan

**Affiliations:** 1 Quantitative Techniques and Operations Management, FORE School of Management, New Delhi, Delhi, India

This article needs to address some important shortcomings before it can be accepted for indexing.

 In Introduction the authors need to clearly state the rationale behind this study, i.e., why do they think that technologies can be useful in the covid time.

 The method section also requires details. See papers on SLR methodology.

 The authors review a huge set of 53 articles, but the discussion is very brief. The authors need to elaborate their discussion by highlighting the key themes and sub-themes. Include a section on gaps in the existing studies and future research directions, which is based on your review (see food waste papers by this reviewer: Chauhan *et al.* 2021a ^1^, Chauhan *et al.* 2021b ^2^). 

 Language check is a must. There are typos and errors, for example, see figure 5 "nmber," etc.

 Best of luck.

Are the rationale for, and objectives of, the Systematic Review clearly stated?

Partly

Is the statistical analysis and its interpretation appropriate?

Not applicable

Are sufficient details of the methods and analysis provided to allow replication by others?

Partly

Are the conclusions drawn adequately supported by the results presented in the review?

Partly

Reviewer Expertise:

Industry 4.0, Circular economy

I confirm that I have read this submission and believe that I have an appropriate level of expertise to confirm that it is of an acceptable scientific standard, however I have significant reservations, as outlined above.

## References

[1] : Technology-Driven Responsiveness in Times of COVID-19: A Fuzzy Delphi and Fuzzy AHP-Based Approach. *International Journal of Global Business and Competitiveness* .2021; 10.1007/s42943-021-00036-6 10.1007/s42943-021-00036-6

[2] : Food loss and waste in food supply chains. A systematic literature review and framework development approach. *Journal of Cleaner Production* .2021;295: 10.1016/j.jclepro.2021.126438 10.1016/j.jclepro.2021.126438

# Response

**Published:** 2021-12-30

**Authors:** Mohammad Nurul Hassan Reza

**Affiliations:** Multimedia University, Malaysia

We would like to express our cordial thanks and gratitude to you for reviewing the manuscript. We believe that the revised version has addressed all the issues. Below are the response to the comments:

**Reviewer's comments:**

 In Introduction the authors need to clearly state the rationale behind this study, i.e., why do they think that technologies can be useful in the covid time.

**Author Response to Comments**

 Thank you very much for your suggestion. The introduction section is further explained to address this issue.

**Reviewer's comments:**

 The method section also requires details. See papers on SLR methodology.

**Author Response to Comments**

 Thank you very much for pointing this out. Due to the word limitation, we planned to make a short SLR using tables and figures to provide the relevant information. However, as the journal advised elaborating relevant sections to respond to the reviewers’ comments, all of the methodological procedures are further explained, including the steps of the article selection process.

**Reviewer's comments:**

 The authors review a huge set of 53 articles, but the discussion is very brief. The authors need to elaborate their discussion by highlighting the key themes and sub-themes.

**Author Response to Comments**

 Thank you very much for your comments. Initially, we were bound to prepare a short SLR due to the limitation of 2500 words. However, the journal has advised elaborating all the relevant sections to address the reviewers’ comments. 

 In the revised version of the manuscript, the discussion section is now improved with two separate tables. Table 3 illustrates the reviewed literature where the potential advantages of Industry 4.0 technologies in supply chain resilience are identified. The reviewed articles are summarized in Table 4, including the technologies of Industry 4.0 that were the main focus of each study. The research area, methodological approach, and findings of each of the studies are also presented. 

 Additionally, categorical analysis is done with a framework illustrating the findings of the study and discussing the themes and sub-themes. In this section, the key enabling technologies of Industry 4.0 in establishing resilient supply chains and the potential advantages (clusters/themes) have been identified. The discussion on the sub-cluster/sub-theme of each cluster/theme is also added, answering the research questions of the study.

**Reviewer's comments:**

 Include a section on gaps in the existing studies and future research directions, which is based on your review.

**Author Response to Comments**

 Thank you very much for the suggestion. A section illustrating the research gaps identified in the reviewed literature and future directions is added.

# Peer review: Reviewer response for version 1

**DOI:** 10.5256/f1000research.76766.r96184

**Published:** 2021-10-21

**Authors:** Lorenzo Ardito

**Affiliations:** 1 Dipartimento di Meccanica Matematica, Management Ringgold Standard Institution, Politecnico di Bari, Bari, Italy

The study is more a bibliometric analysis than a systematic review. A systematic review paper includes a discussion of the articles selected. This article mainly provide some descriptive statistics.

 From these statistics, it is hard to see answers about the two questions. That is, where can I see the "most influential technologies of Industry 4.0 for creating more responsive and resilient supply chains in case of emergencies, such as the COVID-19 outbreak"? Where did you explain "how can the technologies of Industry 4.0 enable supply chains to handle the effects of the COVID-19 outbreak and enhance the responsiveness of the supply chains?" The article must answer these questions delving into the outcomes of the included papers.

 Also, the covid pandemic started at the end of 2019. Why are articles published earlier included?

Are the rationale for, and objectives of, the Systematic Review clearly stated?

Partly

Is the statistical analysis and its interpretation appropriate?

No

Are sufficient details of the methods and analysis provided to allow replication by others?

Partly

Are the conclusions drawn adequately supported by the results presented in the review?

No

Reviewer Expertise:

Innovation, digital transformation

I confirm that I have read this submission and believe that I have an appropriate level of expertise to state that I do not consider it to be of an acceptable scientific standard, for reasons outlined above.

# Response

**Published:** 2021-12-30

**Authors:** Mohammad Nurul Hassan Reza

**Affiliations:** Multimedia University, Malaysia

**Reviewer's comments:**

 The study is more a bibliometric analysis than a systematic review. A systematic review paper includes a discussion of the articles selected. This article mainly provide some descriptive statistics.

 From these statistics, it is hard to see answers about the two questions. That is, where can I see the "most influential technologies of Industry 4.0 for creating more responsive and resilient supply chains in case of emergencies, such as the COVID-19 outbreak"? Where did you explain "how can the technologies of Industry 4.0 enable supply chains to handle the effects of the COVID-19 outbreak and enhance the responsiveness of the supply chains?" The article must answer these questions delving into the outcomes of the included papers.

**Author Response to Comments**

 Thank you very much for your comments. Initially, we were bound to prepare a short SLR due to the limitation of 2500 words. However, the journal has advised elaborating all the relevant sections to address the reviewers’ comments. The revised version addressed all the concerns raised, and further explanation is added in the appropriate sections. 

 In the revised version of the manuscript, the discussion section is now improved with two separate tables. Table 3 illustrates the reviewed literature where the potential advantages of Industry 4.0 technologies in supply chain resilience are identified. The reviewed articles are summarized in Table 4, including the technologies of Industry 4.0 that were the main focus of each study. The research area, methodological approach, and findings of each of the studies are also presented. 

 Additionally, categorical analysis is done with a framework illustrating the findings of the study and answering the research questions. In this section, the key enabling technologies of Industry 4.0 in establishing resilient supply chains and the potential advantages (clusters) have been identified. The discussion on the sub-clusters of each cluster is also added, answering the second research question.

**Reviewer's comments:**

 Also, the COVID pandemic started at the end of 2019. Why are articles published earlier included?

**Author Response to Comments**

 Thank you very much for your suggestion. Initially, we included some papers published before 2019 as the focus of these papers was on Industry 4.0 relating to supply chain disruption. However, to avoid confusion, we have removed the articles published before 2019, and all the figures, tables, and discussions have been revised accordingly. Yet, the authors retained at least three articles focusing on general supply chain disruptions rather than COVID-19, published in 2019. 

 Thank you very much.

# Peer review: Reviewer response for version 1

**DOI:** 10.5256/f1000research.76766.r96185

**Published:** 2021-10-12

**Authors:** Guilherme Brittes Benitez

**Affiliations:** 1 Department of Industrial Engineering, Federal University of Rio Grande do Sul, Porto Alegre, Brazil

I had the opportunity to review the manuscript entitled *“The implications of Industry 4.0 on supply chains amid the COVID-19 pandemic: a systematic review”*. The subject is timely and relevant and the authors did a nice job in their methodological procedures. However, further improvements are necessary for this version to be suitable for indexing.

**Abstract:**

*“Purpose: This research examines the effects of the epidemic on supply chains and how these effects are reduced through Industry 4.0 technology.”* <- Industry 4.0 technologies

*“Findings: This study discusses the impact of COVID-19 on the supply chain and how the emerging technologies of Industry 4.0 can help manufacturers to ease the impact.”* <- this is not a result, this is justificative of your research

*“These technologies will enhance the production system through the automation and optimization of…”* <- “will” is quite odd here. Are you concluding this from the SLR or is this the main finding from your research?

 Originality/value is quite weak. It looks like more a justificative than the real contribution from your work.

**Introduction:**

 Overall, the introduction section is well written and the flow of information sounds good when reading the text. Congrats!

 “In this regard, the role of Industry 4.0 technologies in supply chain management and their revival during COVID-19 is explored in this article.” <- What do you mean by saying “their revival”? It seems out of context here.

**Research method:**

 This section needs improvements. It is too superficial and the authors try to give all the explanations in Table 1 and Figures 1 and 2. This is not sufficient to explain all your methodological procedures. Please, better explain your steps when doing this SLR.

 Why did you consider 2016, 2017, and 2018 in your research given the covid-19 pandemic started in December 2019? From the perspective proposed in the manuscript, you have at least 8 articles you should not include in your analysis.

 You have to better justify why you considered before and pre-pandemic periods.

**Results:**

 The bibliometric analysis is good. I enjoyed seeing how the authors give special attention to this point.

 I believe the authors should explain all images in this section. Despite some images being easy to follow a critical analysis would enrich and benefit the manuscript.

**Discussions:**

 I believe the authors mixed the results and discussions sections. Tables 2 and 3 certainly must be in the Results section and therefore they should be discussed in the discussions section.

 I cannot see the link between Industry 4.0 technologies and each category and its respective advantages. If you create a new column explaining which technologies are related it certainly would help readers. Moreover, you should better present (discuss) these points in your results section.

Are the rationale for, and objectives of, the Systematic Review clearly stated?

Yes

Is the statistical analysis and its interpretation appropriate?

Not applicable

Are sufficient details of the methods and analysis provided to allow replication by others?

Yes

Are the conclusions drawn adequately supported by the results presented in the review?

Partly

Reviewer Expertise:

Industry 4.0 and supply chain.

I confirm that I have read this submission and believe that I have an appropriate level of expertise to confirm that it is of an acceptable scientific standard, however I have significant reservations, as outlined above.

# Response

**Published:** 2021-12-30

**Authors:** Mohammad Nurul Hassan Reza

**Affiliations:** Multimedia University, Malaysia

We would like to thank you for all the valuable comments. We believe that the revised manuscript has addressed all the concerns. Below are the response to the comments:

**Reviewer's comments:**

 “Purpose: This research examines the effects of the epidemic on supply chains and how these effects are reduced through Industry 4.0 technology.” <- Industry 4.0 technologies

**Author Response to Comments**

 Thank you very much for addressing the typo. We have already done the correction.

**Reviewer's comments:**

 “Findings: This study discusses the impact of COVID-19 on the supply chain and how the emerging technologies of Industry 4.0 can help manufacturers to ease the impact.” <- this is not a result, this is justificative of your research.

**Author Response to Comments**

 Thank you very much for pointing this out. The findings are revised as follows:

 Findings: The findings of the study showed that the majority of the articles emphasized the digitalization of supply chain management, acknowledging the fundamentals, applications, and prospects, revealing the drivers and challenges of Industry 4.0 technologies to manage disruptions. Most of the authors identified IoT, big data, cloud computing, additive manufacturing, and blockchain to maintain the supply chain resilience.

**Reviewer's comments:**

 “These technologies will enhance the production system through the automation and optimization of…” <- “will” is quite odd here. Are you concluding this from the SLR or is this the main finding from your research?

**Author Response to Comments**

 Thank you very much for suggesting. We have revised the statement accordingly.

**Reviewer's comments:**

 Originality/value is quite weak. It looks like more a justificative than the real contribution from your work.

**Author Response to Comments**

 We have addressed this and revised it as follows:

 Originality/value: Existing literature on epidemics lacks the basics and practices of utilizing Industry 4.0 technologies in the supply chain recovery process. To fill this research gap, the study summarizes the potential of Industry 4.0 technologies to lessen supply chain disruptions caused by COVID-19. The study findings are valuable for policymakers and practitioners and contribute to supply chain management studies.

**Reviewer's comments:**

 “In this regard, the role of Industry 4.0 technologies in supply chain management and their revival during COVID-19 is explored in this article.” <- What do you mean by saying “their revival”? It seems out of context here.

**Author Response to Comments**

 Thank you very much for noticing this. We have already amended the statement.

**Reviewer's comments:**

 This section needs improvements. It is too superficial and the authors try to give all the explanations in Table 1 and Figures 1 and 2. This is not sufficient to explain all your methodological procedures. Please, better explain your steps when doing this SLR.

**Author Response to Comments**

 Thank you very much for your suggestion. Due to the word limitation, we planned to use tables and figures to provide some information. However, as the journal advised elaborating relevant sections to respond to the reviewers’ comments, all of the methodological procedures are further explained, including the steps of the articles selection process.

**Reviewer's comments:**

 Why did you consider 2016, 2017, and 2018 in your research given the covid-19 pandemic started in December 2019? From the perspective proposed in the manuscript, you have at least 8 articles you should not include in your analysis.

 You have to better justify why you considered before and pre-pandemic periods.

**Author Response to Comments**

 Thank you very much for your suggestion. Initially, we included some papers published before 2019 as the focus of these papers was on Industry 4.0 relating to supply chain disruption. However, to avoid confusion, we have removed the articles published before 2019, and all the figures, tables, and discussions have been revised accordingly. 

**Reviewer's comments:**

 The bibliometric analysis is good. I enjoyed seeing how the authors give special attention to this point.

 I believe the authors should explain all images in this section. Despite some images being easy to follow a critical analysis would enrich and benefit the manuscript.

**Author Response to Comments**

 Thank you very much for your suggestion. Due to the word limitation, we tried to briefly explain the graphs/images. However, as the journal advised to elaborate relevant sections suggested by the reviewers, further explanation is added for each graph/image in the bibliometric analysis.

**Reviewer's comments:**

 I believe the authors mixed the results and discussions sections. Tables 2 and 3 certainly must be in the Results section and therefore they should be discussed in the discussions section.

**Author Response to Comments**

 Thank you very much for addressing the issue. Tables 2 and 3 are updated and inserted in the results section. The core technologies of Industry 4.0 are identified in Table 2, followed by Table 3, demonstrating the advantages of employing these technologies in supply chains during disruptions.

**Reviewer's comments:**

 I cannot see the link between Industry 4.0 technologies and each category and its respective advantages. If you create a new column explaining which technologies are related it certainly would help readers.

 Moreover, you should better present (discuss) these points in your results section.

**Author Response to Comments**

 Thank you very much for the suggestion. We have added Table 3 (Advantages of employing emerging technologies in supply chains.) and Table 4 (Summary of reviewed literature) to address the issue.

 Furthermore, the categorical analysis is done where the prospects of Industry 4.0 technologies in supply chains are identified and classified into clusters and sub-clusters following a thorough interpretation of all the selected articles.
